# Puberty-specific promotion of mammary tumorigenesis by a high animal fat diet

**DOI:** 10.1186/s13058-015-0646-4

**Published:** 2015-11-02

**Authors:** Mark D. Aupperlee, Yong Zhao, Ying Siow Tan, Yirong Zhu, Ingeborg M. Langohr, Erin L. Kirk, Jason R. Pirone, Melissa A. Troester, Richard C. Schwartz, Sandra Z. Haslam

**Affiliations:** Breast Cancer and the Environment Research Program, Department of Physiology, Michigan State University, Biomedical and Physical Sciences Building, Room 2201, 567 Wilson Road, East Lansing, MI 48824 USA; Present address: College of Chemistry and Pharmaceutical Sciences, Qingdao Agricultural University, Qingdao, 266109 People’s Republic of China; Present address: Novartis Institutes for BioMedical Research, 250 Massachusetts Avenue, Cambridge, MA 02139 USA; Department of Pathobiology and Diagnostic Investigation, Michigan State University, East Lansing, MI USA; Present address: Department of Pathobiological Sciences, School of Veterinary Medicine, Louisiana State University, Baton Rouge, LA 70803 USA; Department of Pathology and Laboratory Medicine, University of North Carolina at Chapel Hill, Chapel Hill, NC USA; Lineberger Comprehensive Cancer Center, University of North Carolina at Chapel Hill, Chapel Hill, NC USA; Department of Epidemiology, University of North Carolina at Chapel Hill, Chapel Hill, NC USA; Breast Cancer and the Environment Research Program, Department of Microbiology and Molecular Genetics, Michigan State University, Biomedical and Physical Sciences Building, Room 2201, 567 Wilson Road, East Lansing, MI 48824 USA

## Abstract

**Introduction:**

Increased animal fat consumption is associated with increased premenopausal breast cancer risk in normal weight, but not overweight, women. This agrees with our previous findings in obesity-resistant BALB/c mice, in which exposure to a high saturated animal fat diet (HFD) from peripuberty through adulthood promoted mammary tumorigenesis. Epidemiologic and animal studies support the importance of puberty as a life stage when diet and environmental exposures affect adult breast cancer risk. In this study, we identified the effects of peripubertal exposure to HFD and investigated its mechanism of enhancing tumorigenesis.

**Methods:**

Three-week-old BALB/c mice fed a low-fat diet (LFD) or HFD were subjected to 7,12-dimethylbenz[*a*]anthracene (DMBA)-induced carcinogenesis. At 9 weeks of age, half the mice on LFD were switched to HFD (LFD-HFD group) and half the mice on HFD were switched to LFD (HFD-LFD group). Tumor gene expression was evaluated in association with diet and tumor latency.

**Results:**

The peripubertal HFD reduced the latency of DMBA-induced mammary tumors and was associated with tumor characteristics similar to those in mice fed a continuous HFD. Notably, short-latency tumors in both groups shared gene expression characteristics and were more likely to have adenosquamous histology. Both HFD-LFD and continuous HFD tumors showed similar gene expression patterns and early latency. Adult switch from HFD to LFD did not reverse peripubertal HFD tumor promotion. Increased proliferation, hyperplasia, and macrophages were present in mammary glands before tumor development, implicating these as possible effectors of tumor promotion. Despite a significant interaction between pubertal diet and carcinogens in tumor promotion, peripubertal HFD by itself produced persistent macrophage recruitment to mammary glands.

**Conclusions:**

In obesity-resistant mice, peripubertal HFD is sufficient to irreversibly promote carcinogen-induced tumorigenesis. Increased macrophage recruitment is likely a contributing factor. These results underscore the importance of early life exposures to increased adult cancer risk and are consistent with findings that an HFD in normal weight premenopausal women leads to increased breast cancer risk. Notably, short-latency tumors occurring after peripubertal HFD had characteristics similar to human basal-like breast cancers that predominantly develop in younger women.

**Electronic supplementary material:**

The online version of this article (doi:10.1186/s13058-015-0646-4) contains supplementary material, which is available to authorized users.

## Introduction

Diets high in saturated fat (e.g., a Western diet), as well as high body mass index (BMI) and obesity, have been implicated as risk factors for breast cancer. However, clear delineation of the roles of dietary fat vs. obesity in relation to risk and to breast cancer subtypes is complex (reviewed in [1, 2]). There is increasing consideration of the importance of the time during the life course when diet and/or obesity affect risk. High BMI is associated with decreased risk of estrogen receptor– and progesterone receptor–positive (ER + PR+) premenopausal breast cancers, but it is also associated with increased risk of premenopausal triple-negative breast cancer in African American women [3]. Regardless of race, both luminal ER + PR+ and triple-negative breast cancers are associated with obesity during the postmenopausal period [3]. Emerging evidence indicates that high total fat intake tends to increase breast cancer risk [4]. Notably, increased consumption of animal fat in red meat is associated with increased premenopausal breast cancer risk; importantly, animal fat consumption increased breast cancer in normal weight women but not in overweight and obese women [5]. This report of Farvid et al. [5] is in accord with our previous studies [6], in which we found that a diet high in saturated animal fat (HFD) promoted mammary tumor development in obesity-resistant BALB/c mice. Additional epidemiologic evidence and animal studies support the importance of the pubertal period, a time of rapid breast development, as a time in the life course when diet and environmental exposures can affect breast cancer risk in adulthood. This has important relevance for early-life prevention strategies to reduce breast cancer.

We have investigated the effects of HFD initiated in peripuberty and continued throughout adulthood on the development of carcinogen-induced breast cancers in obesity-resistant BALB/c mice [6]. Using this experimental design, we found that HFD caused significant changes in mammary glands before the development of tumors, such as increased numbers of mammary epithelial hyperplastic lesions, enhanced mammary cell proliferation, increased growth and inflammatory factor gene expression, increased mammary gland chemokine and cytokine gene expression associated with immunosuppressive regulatory T cells, increased vascularization, and elevated numbers of M2 macrophages. Furthermore, HFD dramatically reduced tumor latency, and the early developing tumors exhibited gene expression patterns similar to human basal-like breast cancers. Also noteworthy was the finding that HFD did not cause significant weight gain or obesity or significant changes in blood levels of insulin, glucose, estrogen, or progesterone. Importantly, these findings indicate a potential risk from HFD for a broader segment of the population than only those who become obese, an observation that is consistent with recent epidemiological studies [5].

The present study was undertaken to identify the specific effects of exposure to HFD in peripuberty vs. adulthood, as well as to further investigate the mechanistic basis of dietary animal fat effects that contribute to enhanced tumor development. Using the same animal model, we found that a relatively short exposure to HFD limited to the peripubertal period was sufficient to increase mammary cell proliferation, mammary hyperplasia development, and macrophage recruitment. Further, these effects were sustained and not reversed after changing to a low-fat diet (LFD). Decreased tumor latency was also observed when exposure to HFD was limited to peripuberty. Notably, the early developing tumors exhibited similar gene expression and histopathological characteristics as those observed after continuous HFD exposure. There was also increased occurrence of an ER − PR− phenotype among early developing tumors, regardless of their histopathology. Interestingly, only HFD exposure restricted to adulthood resulted in body weight gain, but this did not have a promotional effect on tumor development compared with continuous exposure to either LFD or HFD. These findings further implicate peripubertal HFD in itself as a potential mammary cancer risk factor.

## Methods

### Animals

Three-week-old female BALB/c mice were purchased from Charles River Laboratories (Portage, MI, USA). Mothers of these mice were maintained on a LabDiet 5L79 diet (LabDiet, St. Louis, MO, USA) before and during pregnancy and while nursing. Upon arrival, mice were randomly distributed into two nonisocaloric diet groups: LFD or HFD. Animals were housed in polysulfone cages and received food and water ad libitum. Food consumption was monitored over a 24-h period weekly, and the weight of food consumed in each diet was similar. Housing facilities were maintained on a 12:12-h light-dark cycle at 20–24 °C with 40–50 % relative humidity. All animal experimentation was conducted in accordance with accepted standards of humane animal care and approved by the All University Committee on Animal Use and Care at Michigan State University.

Diets were initiated at 3 weeks of age. At 9 weeks of age, half the mice on LFD were switched to HFD (LFD-HFD mice, n = 45) and half the mice on HFD were switched to LFD (HFD-LFD mice, n = 42). The remaining mice on HFD (n = 101) or LFD (n = 90) were kept on the same diets for the duration of the experiments. Among the animals in the analyses presented here were mice subjected to 7,12-dimethylbenz[*a*]anthracene (DMBA)-induced tumorigenesis in our initial study comparing continuous HFD with LFD [6]. For all groups, the experimental period ended at 45 weeks of age. The detailed composition of the diets is described in Additional file 1: Table S1.

### Tumorigenesis

Mice were treated with DMBA (Sigma-Aldrich, St. Louis, MO, USA) prepared in vegetable oil and administered by oral gavage (50 mg/kg body weight/mouse) once per week for 4 weeks starting at 5 weeks of age. Additional control mice were kept on the same diet protocols but were not treated with DMBA. Body weights were monitored weekly, and animals were palpated for tumors once per week starting at 8 weeks after the first DMBA dose. Tumor volume was measured twice per week and harvested at 1-cm size. Two hours before being killed, mice were injected with 5-bromo-2′-deoxyuridine (BrdU) (70 μg/g body weight; Sigma-Aldrich) for analysis of cellular proliferation. At termination of all feeding studies, portions of tumors and mammary tissues were either snap-frozen for protein and RNA isolation or formalin-fixed and either processed as whole mounts [7] or paraffin-embedded for hematoxylin and eosin (H&E) staining and immunohistochemistry [8]. Whole-mount preparations of glands and H&E sections were scored for overall morphology and the presence of hyperplasia and neoplasia [9]. All lesions and tumors were reviewed and classified as previously described [10].

### Metabolic parameters

Plasma glucose and insulin levels were metabolic parameters measured as previously described [6]. Nonfasting, randomly sampled glucose and insulin levels were obtained from mice fed ad libitum as an appropriate and acceptable method based on mouse feeding habits and the stress caused by fasting [11]. Plasma levels of glucose were determined by ACCU-CHEK Compact glucometer (Roche Diagnostics, Indianapolis, IN, USA), and insulin levels were determined with an insulin enzyme-linked immunosorbent assay kit from EMD Millipore (catalog number EZRMI-13K; Billerica, MA, USA), following the manufacturer’s instructions.

### Immunofluorescence and immunohistochemical analyses

Detection of ERα, PR, and HER2/Neu was performed as previously described [6]. ERα was detected with mouse anti-ERα [1:10 in phosphate-buffered saline (PBS)–0.5 % Triton X-100, catalog number NCL-ER-6F11; Leica Biosystems, Newcastle upon Tyne, UK) followed by Alexa Fluor 488–labeled goat anti-mouse secondary antibody (Ab) (1:200 in PBS; Life Technologies, Grand Island, NY, USA). PR was detected with rabbit anti-PR (1:200 in 2 % bovine serum albumin in phosphate-buffered saline (PBSA), catalog number A0092; DAKO, Carpinteria, CA, USA) followed by Alexa 488–labeled goat anti-rabbit secondary Ab (1:200 in PBS; Life Technologies). For HER2/Neu, sections were not blocked but immediately incubated with goat anti-Neu (1:50 in PBS, catalog number sc-284-G; Santa Cruz Biotechnology, Santa Cruz, CA, USA) followed by incubation with an Alexa Fluor 488–labeled donkey anti-goat secondary Ab (1:400 in PBS; Life Technologies). For ERα and HER2/Neu assessment, a minimum of 1000 cells were counted for each tumor. Tumors were considered to be ERα-positive (ER+) if 10 % or more of the total cells counted were ER+ [12]. A minimum of 500 cells per section for each tumor were counted.

β-catenin was detected with rabbit polyclonal anti-β-catenin (1:200 in 1 % PBSA, catalog number C2206-1 ml; Sigma-Aldrich) at 4 °C overnight, followed by Alexa Fluor 488–labeled goat anti-rabbit secondary Ab (1:400 in PBS; Life Technologies). To analyze overall β-catenin fluorescence intensity, the average pixel intensity of all positively stained cells within the ductal epithelium was determined. A threshold was set to exclude background fluorescence, and images were gated to include intensity measurements only from positively staining epithelial cells. To assess nuclear localization of β-catenin, a nuclear β-catenin score between 1 and 5 was assigned to each tumor by two independent evaluators, where 1 was the absence of nuclear β-catenin and 5 was more than 50 % of cells expressing nuclear β-catenin.

Analysis of macrophages in mammary gland or tumor sections was performed as previously described [6]. Macrophages were detected with rat monoclonal anti-F4/80 (1:75 in PBS–0.5 % Triton X-100, catalog number MCA497R; AbD Serotec, Raleigh, NC, USA), followed by incubation with Alexa 488–labeled goat anti-rat secondary Ab (1:100 in PBS; Life Technologies). M2-activated macrophages were detected by double-labeling with rat monoclonal anti-F4/80 and goat anti-arginase 1 (anti-Arg1) (1:200 in PBS–0.5 % Triton X-100, catalog number sc-18354; Santa Cruz Biotechnology), followed by appropriate secondary antibodies conjugated with fluorescent labels. The number of F4/80 and/or Arg1-positive cells was expressed as cells per structure in the mammary gland periepithelial area and cells per image in tumor samples.

Detection of BrdU incorporation was used as a measure of proliferation as previously described [6]. BrdU incorporation was detected with mouse anti-BrdU (1:100 in PBS–0.5 % Triton X-100, catalog number ab27958; Abcam, Cambridge, MA, USA), followed by Alexa Fluor 488–labeled goat anti-mouse secondary Ab (1:200 in PBS; Life Technologies). A minimum of 1000 cells were counted for each section, and a minimum of 2–3 tissue sections per animal were analyzed.

All immunofluorescence sections were counterstained with 4′,6-diamidino-2-phenylindole. The stained sections were visualized with a Nikon Eclipse TE2000-U fluorescence microscope (Nikon, Melville, NY, USA) using a 40× lens objective, and the captured fluorescence images were analyzed using MetaMorph software (Molecular Devices, Sunnyvale, CA, USA). Histological sections of mammary glands stained for macrophages and cellular proliferation were analyzed by mammary gland epithelial structure: ducts or hyperplastic foci. Hyperplastic structures had multiple cell layers of noticeably distorted epithelium compared with normal epithelial structures.

CD31 staining was used to detect blood vessels as previously described [6]. CD31 was detected with rabbit anti-CD31 (1:50 in PBS–0.5 % Triton X-100, catalog number AP15436PU-N; Acris Antibodies, San Diego, CA, USA), followed by secondary swine anti-rabbit Ab (DAKO) and VECTASTAIN ABC reagent (PK-7100; Vector Laboratories, Burlingame, CA, USA). The sections were then incubated with metal-enhanced 3,3′-diaminobenzidine substrate solution (1:10 dilution with Pierce stable peroxide substrate buffer; Thermo Scientific, Rockford, IL, USA) and counterstained with hematoxylin. The stained sections were visualized with a Nikon Eclipse E400 light microscope (Nikon) using a 40× lens objective. A minimum of 1000 cells were counted for each section, and a minimum of 2–3 tissue sections per animal were analyzed. Digital micrographs were captured and quantified as previously described [6]. Blood vessel density was expressed as the percentage of CD31-positive squares.

### Quantitative reverse transcription polymerase chain reaction analysis

Total RNA was isolated from mouse inguinal mammary glands or tumors, cDNAs were prepared, and quantitative reverse transcription polymerase chain reaction (qRT-PCR) was performed as previously described [6]. Primers for the following selected RNAs were purchased from SABiosciences (Frederick, MD, USA): transforming growth factor α (Tgfa) (PPM03051G), chemokine (C-C motif) ligand 1 (Ccl1) (PPM03138C), Ccl17 (PPM02963B), Ccl20 (PPM03142B), Ccl22 (PPM02950B), transforming growth factor β1 (Tgfb1) (PPM02991B), neurotrophin 3 (Ntf3) (PPM04325A), transformation-related protein 53 (Trp53) (PPM02931C), cyclin D2 (Ccnd2) (PPM02900F), catenin (cadherin associated protein), beta 1 (Ctnnb1) (PPM03384A), breast cancer 1, early onset (Brca1) (PPM03442A), apoptotic peptidase activating factor 1 (Apaf1) (PPM03407F), bone morphogenetic protein 7 (Bmp7) (PPM03001C), Bmp10 (PPM04457A), hypoxanthine-guanine phosphoribosyltransferase (PPM03559F), and heat shock protein 90 alpha (cytosolic), class B member 1 (PPM04803F). The RNAs analyzed for each treatment group are presented in Additional file 2: Table S2.

### Microarray analysis

Agilent Technologies (Santa Clara, CA, USA) 4 × 44K whole mouse genome microarrays were performed according to the manufacturer’s protocol with linear amplification and two-color hybridization using total RNA isolated from mouse mammary tumors (Additional file 2: Table S2). The reference channel was universal mouse reference (as described in [13]) and was labeled with cyanine 5. Spots that had intensity greater than 10 dpi in at least 80 % of samples were selected for subsequent analysis. Data were Lowess-normalized, and missing data were imputed using *k*-nearest neighbors with *k* = 10. A total of 25 microarrays were analyzed. Two-class significance analysis of microarrays was performed to identify differentially expressed genes between early vs. late tumor onset and HFD vs. LFD. All statistical analyses were conducted in R using the limma package in Bioconductor. For genes significantly associated (*p* < 0.05) with early vs. late tumor onset, gene ontology analyses were conducted using Ingenuity Pathway Analysis (QIAGEN, Redwood City, CA, USA). The data discussed in this publication have been deposited in the National Center for Biotechnology Information Gene Expression Omnibus (GEO) [14] database and are accessible at accession number [GEO:GSE73983] [15].

### Statistical analyses

qRT-PCR assays were statistically analyzed using proprietary software from SABiosciences. Otherwise, the results were expressed as mean ± standard error of the mean. Differences were considered significant at *p* < 0.05 using Student’s *t* test or analysis of variance followed by a Tukey’s multiple-comparisons test, as appropriate. Tumor incidence was analyzed by the χ^2^ test. Tumor latencies were determined from Kaplan-Meier plots.

## Results

### Peripubertal exposure to high-fat diet promotes tumorigenesis

**Fig. 1:**
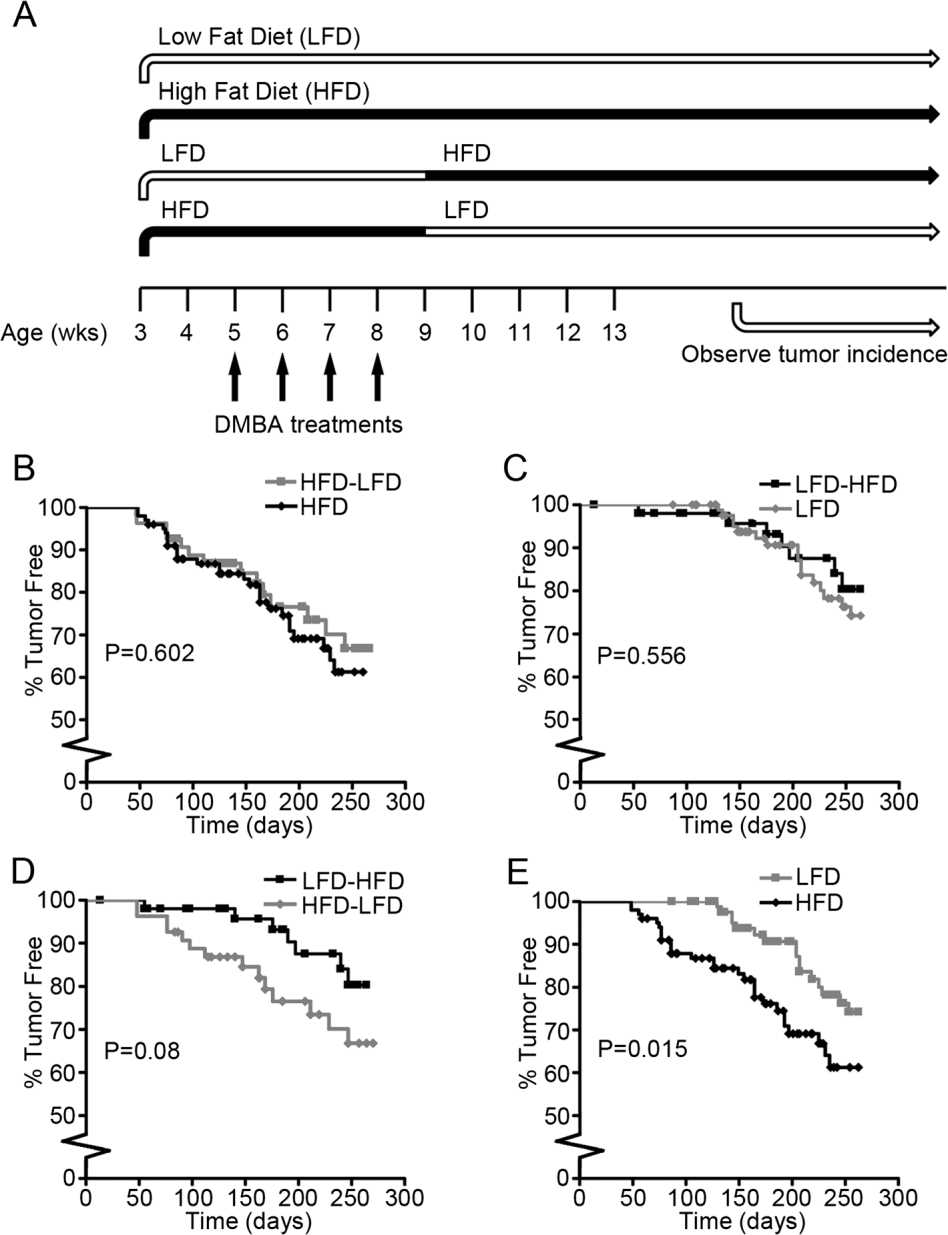
Experimental design and Kaplan-Meier plot of 7,12-dimethylbenz[*a*]anthracene (DMBA)-induced mammary tumors. **a** BALB/c mice were started on a high saturated animal fat diet (HFD) or a low-fat diet (LFD) at 3 weeks old. Mice were switched from HFD to LFD and from LFD to HFD at 9 weeks old. DMBA was administered weekly from ages 5 to 8 weeks. Tumor incidence was observed. **b**–**e** Kaplan-Meier plots of tumor incidence. Time = number of days after last DMBA treatment (HFD mice, n = 101; LFD mice, n = 90; HFD-LFD mice, n = 42; LFD-HFD mice, n = 45). **b** HFD-LFD vs. HFD. **c** LFD-HFD vs. LFD. **d** LFD-HFD vs. HFD-LFD. **e** LFD vs. HFD

Mice were fed HFD or LFD during peripuberty, from 3 to 9 weeks of age, and then switched to LFD (HFD-LFD) or HFD (LFD-HFD), respectively, for the remainder of the experimental period up to 45 weeks of age. Two additional groups of mice were fed HFD or LFD starting at 3 weeks of age and continuing to 45 weeks of age (Fig. 1a). Kaplan-Meier plots show similar tumor incidence and latency patterns for the HFD-LFD and continuous HFD (HFD) groups (Fig. 1b), as well as for the continuous LFD (LFD) and LFD-HFD groups (Fig. 1c). Although the trends of increased incidence and decreased latency of the HFD-LFD group did not reach statistical significance compared with the LFD-HFD group (Fig. 1d), the overall pattern was similar to continuous HFD vs. continuous LFD (Fig. 1e). The Kaplan-Meier plots for LFD vs. LFD-HFD and HFD vs. HFD-LFD were almost identical and not statistically different (Fig. 1b, c). These results indicate that the short period of 6 weeks on HFD during the peripubertal period (HFD-LFD group) had effects on tumor incidence and latency similar to those of mice fed continuously on HFD from puberty through adulthood up to 45 weeks of age. Furthermore, the switch from HFD to LFD in adulthood did not reverse the effects of peripubertal HFD on tumor promotion.

### Tumor characteristics

**Fig. 2:**
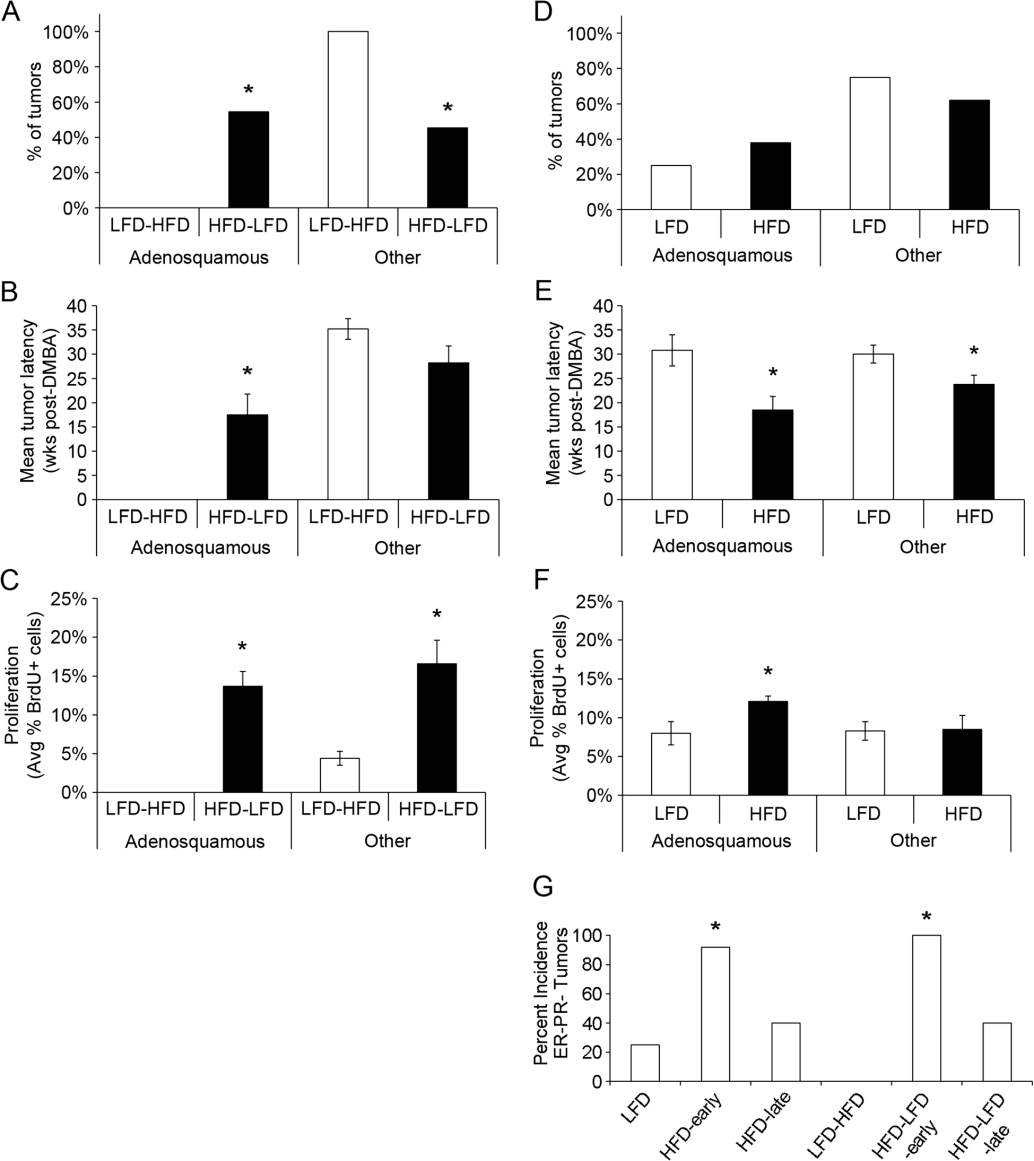
Tumor characteristics based on histopathology. **a** Mice that were on the high saturated animal fat diet (HFD) and were switched to the low-fat diet (HFD-LFD) had an increased proportion of adenosquamous tumors compared with mice on the HFD that were switched to LFD (LFD-HFD) (HFD-LFD 55 % vs. LFD-HFD 0 %). *p < 0.05. Conversely, LFD-HFD-fed mice had an increased proportion of nonadenosquamous tumors compared with HFD-LFD-fed mice (LFD-HFD 100 % vs. HFD-LFD 45 %). *p < 0.05. **b** Adenosquamous tumors (n = 6) in HFD-LFD-fed mice had reduced latency compared with other tumor types (n = 5). *p < 0.05. **c** Both adenosquamous and other mammary tumor types had increased proliferation in HFD-LFD-fed mice compared with tumors in LFD-HFD-fed mice (n = 6). *p < 0.05. **b** and **c** *Bars* represent mean ± standard error of the mean (SEM). **d** HFD-fed (n = 29 tumors) and LFD-fed (n = 16 tumors) mice had a similar incidence of all tumor types. **e** HFD-fed mice had reduced tumor latency for both adenosquamous (n = 12) and other tumor types (n = 17) compared with total tumors in LFD-fed mice (adenosquamous, n = 5; other, n = 11). *p < 0.05. **f** Adenosquamous tumors from HFD-fed mice had increased proliferation compared with all other tumors in HFD- and LFD-fed mice. *p < 0.05. **b**, **c**, **e**, and **f** *Bars* represent mean ± SEM. **g** Incidence of estrogen receptor– and progesterone receptor–negative (ER − PR−) tumors was increased among HFD-early (n = 11 of 12) and HFD-LFD-early (n = 6 of 6) tumors compared with LFD (n = 4 of 16), HFD-late (n = 2 of 5), LFD-HFD (n = 0 of 6), and HFD-LFD-late (n = 2 of 5) tumors. *p < 0.05. *BrdU* 5-bromo-2′-deoxyuridine, *DMBA* 7,12-dimethylbenz[*a*]anthracene

Analysis of the tumor characteristics of histopathology, time to tumor development, and receptor status (Table 1) showed that 6 mice in the HFD-LFD group (n = 11) developed tumors less than 23 weeks after DMBA exposure, before any tumor incidence in the LFD-HFD group. These tumors were almost exclusively adenosquamous carcinomas and ER−, PR−, and Her2−. This is in contrast to the majority of tumors that developed later (≥23 weeks after DMBA exposure) in the LFD-HFD and HFD-LFD groups. There was no significant difference in the incidence proportion of ER+ and ER− glandular, cribriform, or papillary carcinomas between the later-developing tumors in the HFD-LFD and LFD-HFD groups (i.e., HFD-LFD late tumors, 3 of 5 ER+; LFD-HFD tumors, 4 of 6 ER+). The overall proportion of adenosquamous tumors in the HFD-LFD group was significantly higher than in the LFD-HFD group (*p* < 0.05) (Fig. 2a). The tumor latency of adenosquamous tumors was significantly shorter than that for all other tumor types in the HFD-LFD and LFD-HFD groups (*p* < 0.05) (Fig. 2b). The latency of nonadenosquamous tumors did not differ significantly between the HFD-LFD and LFD-HFD groups. Analysis of tumor cell proliferation showed that tumors in the HFD-LFD group collectively, as well as adenosquamous tumors specifically, exhibited significantly higher proliferation than tumors in the LFD-HFD group (*p* < 0.05) (Fig. 2c). A similar analysis was carried out among continuous HFD and LFD groups. There were no significant differences in the percentage of adenosquamous or other tumor types between the two diets (Fig. 2d). However, the HFD adenosquamous tumors had a significantly shorter latency (Fig. 2e) and significantly higher cell proliferation (Fig. 2f) than adenosquamous tumors that arose in mice on LFD (*p* < 0.05). There was equal representation of high-grade epithelial tumors across all diet treatments and early vs. late tumors (data not shown).

**Table 1:** Tumor characteristics

Diet	Weeks after first DMBA treatment	Histopathology	Hormone receptor status^a^
LFD-HFD	23	Glandular/spindle cell carcinoma	ER−PR−
	28	Papillary carcinoma	ER−PR−
	31	Glandular carcinoma	ER+PR+
	37	Glandular carcinoma	ER+PR+
	38	Papillary carcinoma	ER+PR+
	42	Glandular carcinoma	ER+PR+
HFD-LFD-early	10	Adenosquamous carcinoma	ER−PR−
	10	Adenosquamous carcinoma	ER−PR−
	16	Adenosquamous carcinoma	ER−PR−
	14	Adenosquamous carcinoma	ER−PR−
	17	Adenosquamous carcinoma	ER−PR−
	19	Cribriform carcinoma	ER−PR−
HFD-LFD-late	26	Papillary carcinoma	ER−PR−
	28	Papillary carcinoma	ER+PR+
	33	Glandular carcinoma	ER+PR+
	35	Glandular carcinoma	ER+PR+
	38	Adenosquamous carcinoma	ER−PR−

*DMBA* 7,12-dimethylbenz[*a*]anthracene, *ER* estrogen receptor, *HFD* high saturated animal fat diet, *LFD* low-fat diet, *PR* progesterone receptor

^a^ER status was based on >10 % receptor-positive cell. All ER−PR− tumors that were tested were also Her2−

There were notable similarities between tumors developing on continuous HFD and on HFD restricted to peripuberty. Namely, both groups had early-onset adenosquamous tumors (reduced latency <23 weeks following exposure) and increased cell proliferation. Tumors with other histopathologies (i.e., glandular, cribriform, and papillary carcinomas) also showed significantly reduced latency in mice on continuous HFD compared with those on LFD (*p* < 0.05) (Fig. 2e), indicating that HFD also affects onset of these tumor types. These effects on continuous HFD-fed animals suggest that, although the peripubertal window may be especially sensitive, the effects of HFD persist in later adulthood. Furthermore, short-latency tumors occurring in both HFD and HFD-LFD mice showed a higher incidence of an ER−PR− phenotype than tumors in all other treatment groups (Fig. 2g). All ER−PR− tumors (Table 1) tested for Her2 were negative for that marker (data not shown).

**Fig. 3:**
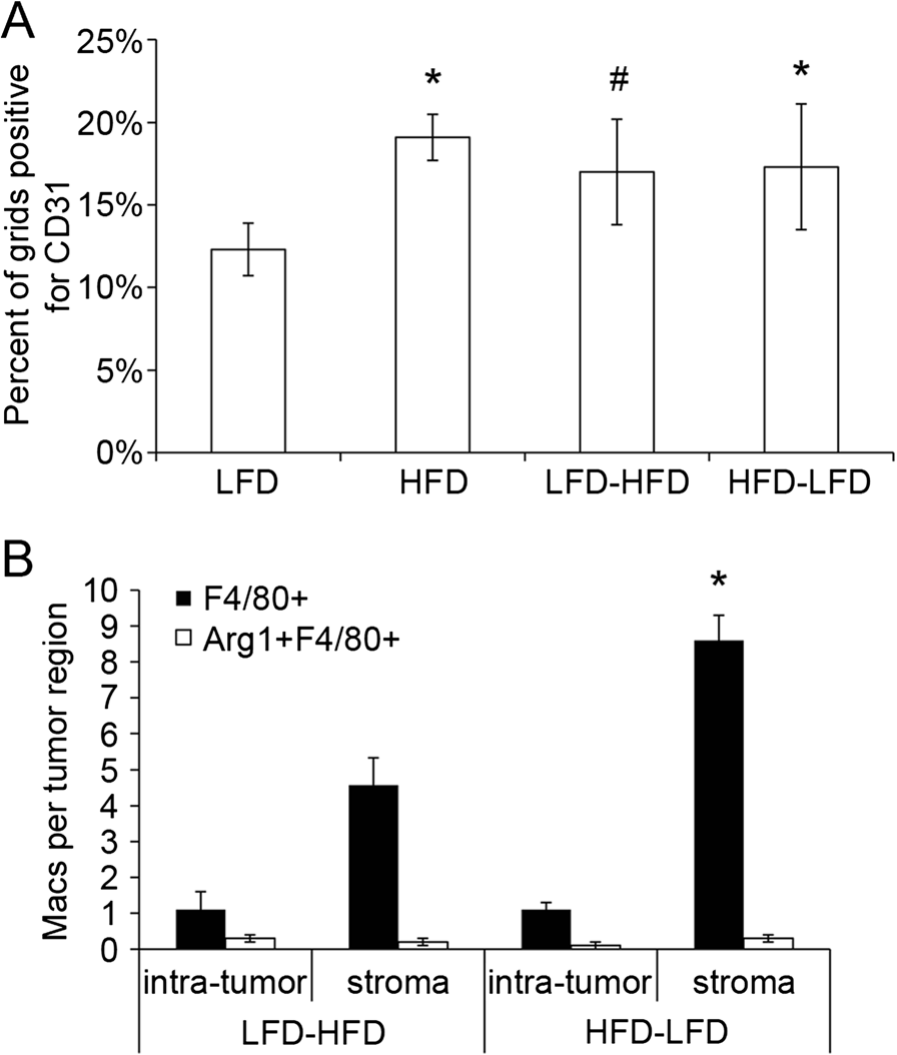
Overall tumor characteristics in HFD-, LFD-, HFD-LFD-, and LFD-HFD-fed mice. **a** Blood vessel density (CD31 staining) was increased in LFD-HFD-fed murine mammary tumors (#*p* = 0.07) and significantly increased in HFD- and HFD-LFD-fed murine mammary tumors compared with LFD-fed murine mammary tumors. *p < 0.05. **b** Macrophage (Macs; F4/80 staining) recruitment was increased within the stroma of tumors from mice fed a HFD-LFD vs. LFD-HFD. *p < 0.05. Bars represent mean ± standard error of the mean; n = 4–10 tumors per diet treatment. *Arg1* arginase 1, *HFD* high saturated animal fat diet, *HFD-LFD* mice on high saturated animal fat diet switched to low-fat diet, *LFD* low-fat diet, *LFD-HFD* mice on low-fat diet switched to high saturated animal fat diet

We previously determined that continuous HFD treatment resulted in short-latency tumors that also had significant changes in angiogenesis and macrophage recruitment [6]. Thus, we analyzed these same properties in all tumors that developed in the HFD-LFD and LFD-HFD groups, and we compared the results to all tumors obtained with continuous diet treatments. Tumors from HFD-LFD mice had significantly increased angiogenesis compared with LFD tumors, similar to that observed in continuous HFD tumors (Fig. 3a). There was also a trend toward increased angiogenesis in LFD-HFD tumors (*p* = 0.07). Analysis of macrophage localization in tumors showed that, compared with LFD-HFD tumors, there were significantly increased numbers of macrophages within the stroma of HFD-LFD tumors (Fig. 3b). There was no difference in the level of Arg1-positive, alternatively activated macrophages between HFD-LFD and LFD-HFD tumors (Fig. 3b). Taken together, these results indicate that increased angiogenesis and macrophage recruitment were likely contributors to tumor promotion in HFD-LFD mice. Interestingly, because LFD-HFD tumors showed trends toward increased angiogenesis and macrophage recruitment, this suggests that when HFD exposure is limited to adulthood these tumor characteristics are also affected, although to a lesser extent than when HFD is limited to peripubertal exposure.

### Microarray analysis of gene expression in tumors

**Fig. 4:**
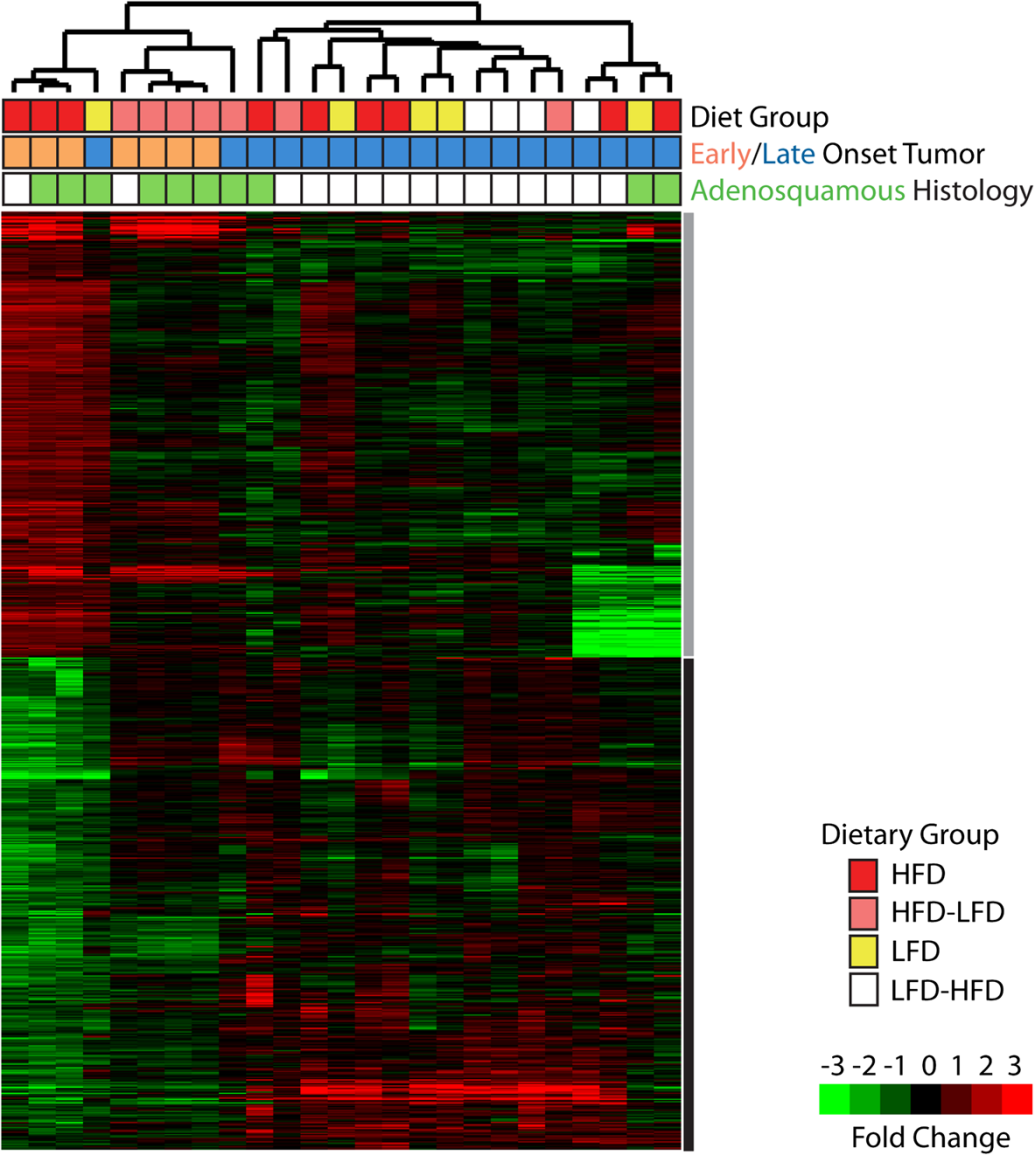
Microarray heat map cluster analysis. The heat map and dendrogram represent a two-class significance analysis of genes differentially expressed between early vs. late tumor onset and high saturated animal fat diet (HFD) vs. a low-fat diet (LFD). Two gene clusters were identified: one enriched for genes upregulated among early-occurring tumors (*gray bar*) and one enriched for genes downregulated among early-occurring tumors (*black bar*). Early-occurring tumors from HFD- and HFD-LFD-fed mice cluster together. Diet group, early vs. late tumor onset, and adenosquamous vs. other histologies are noted. *HFD-LFD* mice on high saturated animal fat diet switched to low-fat diet, *LFD-HFD* mice on low-fat diet switched to high saturated animal fat diet

Microarray analysis was performed to examine differential patterns of gene expression between early- and late-occurring tumors that arose in the mice fed HFD, LFD, HFD-LFD, LFD-HFD. Comparison of tumors that arose in mice fed continuous HFD (n = 9) vs. those fed continuous LFD (n = 3) yielded no significant difference in their patterns of gene expression (data not shown), suggesting that the effects of diet on tumor characteristics were indirect. That is, diet affected tumorigenesis by decreasing latency, with resulting changes in gene expression for early- vs. late-onset tumors. Comparison of animals with early tumors (n = 7) vs. late tumors (n = 18) yielded 770 genes. A hierarchical cluster of these genes resulted in two main sample clusters (Fig. 4). The first cluster (upregulated in early-onset tumors) was enriched, but not exclusively, for adenosquamous histology and, importantly, for both continuous HFD and peripubertal HFD (HFD-LFD) vs. continuous LFD and adult HFD (LFD-HFD). This reinforces the conclusion that peripubertal exposure to HFD is sufficient to promote tumorigenesis similarly to continuous exposure to HFD [6].

In ontology analyses (Additional file 3: Table S3), we identified several statistically significant (i.e., *p* < 0.05) canonical pathways altered in the short latency tumors that cluster together. Upregulated pathways included those involved in cell cycle regulation (i.e., G_1_/S checkpoint regulation, G_2_/M DNA damage checkpoint regulation, cyclins and cell cycle regulation, antiproliferative role of TOB in T cell signaling, cell cycle control of chromosomal replication) and organismal growth [i.e., mechanistic target of rapamycin (mTOR) signaling, purine nucleotides de novo biosynthesis II], genotoxic stress [i.e., growth arrest and DNA damage 45 (GADD45) signaling, eukaryotic initiation factor 2 (EIF2) signaling], and molecular mechanisms of cancer. Downregulated pathways included those involved in suppressing proliferation and/or increasing apoptosis of breast cancer cells [i.e., farnesoid X receptor (FXR)/retinoid X receptor (RXR) activation, liver X receptor (LXR)/RXR activation) and classical inflammatory processes (i.e., coagulation system, acute phase response signaling).

### Expression analysis of genes associated with HFD promotion of tumor development

We performed qRT-PCR for expression of RNAs that were previously reported to be either significantly upregulated or downregulated in short latency mammary tumors that developed in continuous HFD mice (HFD-early) [6]. Results were compared for fold changes of HFD-early and HFD-late tumors vs. LFD tumors (Table 2) and of HFD-LFD-early and HFD-LFD-late tumors vs. LFD-HFD tumors (Table 3). HFD-early and HFD-late tumors were distinguishably different from each other. HFD-LFD-early tumors showed significant upregulation of RNAs for *Ntf3*, *Trp53*, *Ccnd2*, *Ctnnb1*, *Brca1*, *Apaf1* and *Bmp7*, as well as downregulation of *Bmp10*. This showed excellent concordance with our previously published observations for these genes in HFD-early tumors [6] (see HFD-early in Table 2). HFD-LFD-late tumors showed a pattern of expression among these genes more similar to that in LFD-HFD tumors, and distinguishably different from that in the early occurring HFD-LFD tumors. Compared with HFD-LFD-early tumors, quite modest increases were observed for *Ccnd2*, *Ctnnb1*, and *Brca1* expression in the HFD-LFD-late tumors, while *Trp53* expression was modestly reduced. No significant alterations in expression were observed for the other genes. These results confirm that the HFD-LFD-early tumors retain the same basal-like gene expression pattern observed for the HFD-early tumors in our previous study [6].

**Table 2:** Gene expression in continuous diet DMBA-induced tumors

		Fold regulation (compared with LFD)	
Symbol	Description	HFD-early^a^	HFD-late
*Ntf3*	Neurotrophin 3	53.4	6.3
*Trp53*	Transformation related protein 53	1.6	NS
*Ccnd2*	Cyclin D2	3.5	3.5
*Ctnnb1*	Catenin (cadherin associated protein), beta 1	1.8	−3.6
*Brca1*	Breast cancer 1	1.8	−1.5
*Apaf1*	Apoptotic peptidase activating factor 1	1.8	1.3
*Bmp7*	Bone morphogenetic protein 7	3.2	NS
*Bmp10*	Bone morphogenetic protein 10	−3.4	NS

*DMBA* 7,12-dimethylbenz[*a*]anthracene *HFD* high saturated animal fat diet, *LFD* low-fat diet

*p* < 0.05 for all genes listed, except NS = no significant change [n = 4 tumors per diet group (LFD, HFD-early, HFD-late)]

^a^HFD-early data from Zhao et al. [6]

**Table 3:** Gene expression in switched diet DMBA-induced tumors

		Fold regulation (compared with LFD-HFD)	
Symbol	Description	HFD-LFD-early	HFD-LFD-late
*Ntf3*	Neurotrophin 3	70.0	NS
*Trp53*	Transformation related protein 53	1.5	−1.2
*Ccnd2*	Cyclin D2	12.4	3.1
*Ctnnb1*	Catenin (cadherin associated protein), beta 1	5.4	1.4
*Brca1*	Breast cancer 1	2.9	1.4
*Apaf1*	Apoptotic peptidase activating factor 1	1.4	NS
*Bmp7*	Bone morphogenetic protein 7	5.6	NS
*Bmp10*	Bone morphogenetic protein 10	−2.7	NS

*DMBA* 7,12-dimethylbenz[*a*]anthracene *HFD* high saturated animal fat diet, *HFD-LFD*, mice on high saturated animal fat diet switched to low-fat diet, *LFD* low-fat diet, *LFD-HFD* mice on low-fat diet switched to high saturated animal fat diet

*p* < 0.05 for all genes listed, except NS = no significant change [n = 4 tumors per diet group (LFD-HFD, HFD-LFD-early, HFD-LFD-late)]

### Analysis of dietary effects on mammary glands before tumor development

**Fig. 5:**
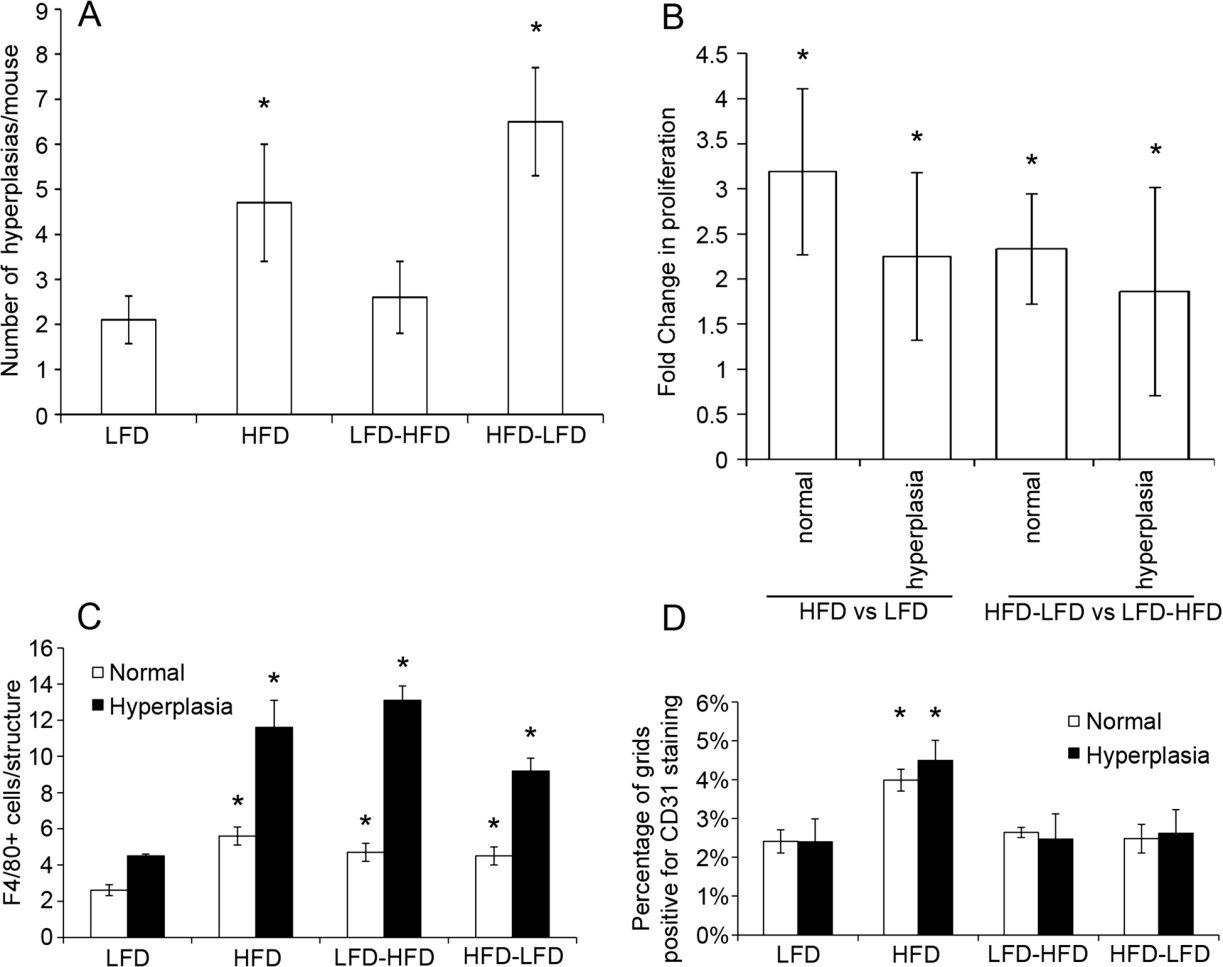
Effect of various diets on mammary glands at 13 weeks of age and before tumor development. **a** More hyperplastic lesions (hyperplasias) developed in HFD- and HFD-LFD-fed, 7,12-dimethylbenz[*a*]anthracene-treated mice. *p < 0.05. **b** Mice fed HFD or HFD-LFD exhibited increased cellular proliferation in both normal epithelium and hyperplastic lesions (hyperplasia) compared with those fed LFD or LFD-HFD, respectively, as measured by 5-bromo-2′-deoxyuridine incorporation. *p < 0.05. **c** Macrophage (F4/80-stained cells) recruitment was increased adjacent to normal ducts and hyperplastic lesions (hyperplasia) (*p < 0.05) in mammary glands of HFD-, HFD-LFD-, and LFD-HFD-fed mice compared with those of LFD-fed mice. **d** Blood vessel density (CD31 staining) was significantly increased adjacent to normal mammary gland structures and hyperplastic lesions (hyperplasia) (*p < 0.05) in HFD-fed compared with LFD-, HFD-LFD-, or LFD-HFD-fed mice. Bars represent mean ± standard error of the mean (n = 5 mice per diet group for each assay). *HFD* high saturated animal fat diet, *HFD-LFD* mice on high saturated animal fat diet switched to low-fat diet, *LFD* low-fat diet, *LFD-HFD* mice on low-fat diet switched to high saturated animal fat diet

Because 6 weeks on HFD (i.e., HFD-LFD) was sufficient to promote tumor development similar to continuous HFD, we were interested in analyzing the effects of HFD before tumor development to elucidate potential underlying mechanisms. We had previously noted that increased numbers of mammary hyperplastic lesions, increased angiogenesis, and increased macrophage recruitment were associated with increased tumor incidence and reduced latency in mice fed a continuous HFD [6]. Thus, we analyzed these same factors in HFD-LFD and LFD-HFD mammary glands at 4 weeks after diet switches. These glands were taken from mice of the same age (13 weeks of age) for comparison with continuous diet analyses (Fig. 5). The number of epithelial hyperplastic lesions was significantly greater in HFD-LFD mammary glands than in LFD-HFD mammary glands (Fig. 5a). Proliferation in normal glandular structures and hyperplastic lesions was significantly increased in HFD-LFD compared with LFD-HFD mammary glands, similarly to continuous HFD compared with continuous LFD mammary glands (Fig. 5b). Macrophage recruitment to the periepithelial mammary stroma of glandular structures and hyperplastic lesions was significantly increased for both HFD-LFD and LFD-HFD mammary glands compared with continuous LFD mammary glands (Fig. 5c) and similar to that previously reported for continuous HFD mammary glands [6]. However, neither HFD-LFD nor LFD-HFD increased angiogenesis in mammary glands compared with continuous LFD, as was the case with continuous HFD mice (Fig. 5d).

### HFD modulation of mammary gland gene expression before tumor development

To gain insight into potentially specific effects of peripubertal HFD vs. adult HFD exposures in tumor development, we analyzed RNA expression by qRT-PCR of genes that we had previously reported to be either significantly upregulated or downregulated by continuous HFD in mammary glands before tumor development [6]. The results were compared for fold changes between HFD, HFD-LFD, and LFD-HFD vs. LFD. The RNA levels of *Tgfa*, *Tgfb1*, and the chemokines *Ccl1*, *Ccl17*, *Ccl20*, and *Ccl22* were analyzed (Table 4). HFD, HFD-LFD, and LFD-HFD mammary glands all exhibited significant, approximately twofold increases in *Tgfa* RNA compared with LFD mammary glands. No significant changes were observed in the RNA levels of *Tgfb1* and the other assayed chemokines in HFD-LFD mammary glands; however, these RNAs were upregulated in LFD-HFD mammary glands to a similar extent as in HFD mammary glands. Comparisons made for HFD-LFD vs. LFD-HFD were consistent with these results (data not shown). This suggests an association with adult HFD and not with the peripubertal HFD promotional window. Furthermore, these results suggest that both peripubertal-only and adult-only HFD exposure increase *Tgfa* RNA levels, similarly to continuous HFD exposure.

**Table 4:** RT-PCR analysis of gene expression at 13 weeks old with DMBA treatment

		Fold regulation (compared with LFD)		
Symbol	Description	HFD	LFD-HFD	HFD-LFD
*Tgfa*	Transforming growth factor alpha	2.4	2.1	2.0
*Ccl1*	Chemokine (C-C motif) ligand 1	3.8	3.2	NS
*Ccl17*	Chemokine (C-C motif) ligand 17	2.8	4.0	NS
*Ccl20*	Chemokine (C-C motif) ligand 20	11.0	5.0	NS
*Ccl22*	Chemokine (C-C motif) ligand 22	4.0	6.1	NS
*Tgfb1*	Transforming growth factor, β 1	1.6	2.2	NS

*DMBA* 7,12-dimethylbenz[*a*]anthracene *HFD* high saturated animal fat diet, *HFD-LFD*, mice on high saturated animal fat diet switched to low-fat diet, *LFD* low-fat diet, *LFD-HFD* mice on low-fat diet switched to high saturated animal fat diet, *RT-PCR* reverse transcription polymerase chain reaction

*p* < 0.05 for all genes listed, except NS = no significant change (n = 4 mice per diet treatment)

### Immunofluorescence determination of β-catenin expression associated with HFD promotion of tumor development

**Fig. 6:**
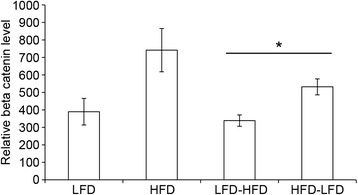
β-catenin regulation by diet treatment in mammary glands at 13 weeks of age. Before tumor development, β-catenin levels were measured based on immunofluorescence. β-catenin levels were increased by HFD (*p* = 0.08) and HFD-LFD. *p < 0.05. *Bars* represent mean ± standard error of the mean (n = 5 mice per diet treatment). *HFD* high saturated animal fat diet, *HFD-LFD* mice on high saturated animal fat diet switched to low-fat diet, *LFD* low-fat diet, *LFD-HFD* mice on low-fat diet switched to high saturated animal fat diet

Previously, we identified basal-like breast cancer gene expression characteristics in our gene ontology analysis of HFD early tumors [6]. The key genes identified were elements of the β-catenin signaling pathway, including β-catenin itself. Analysis of β-catenin expression by immunofluorescence in pretumor mammary glands showed increased expression in HFD-LFD mice (approximately 1.5-fold, *p* < 0.05) and a trend toward increased expression in continuous HFD mice (approximately twofold; *p* = 0.08) (Fig. 6).

**Fig. 7:**
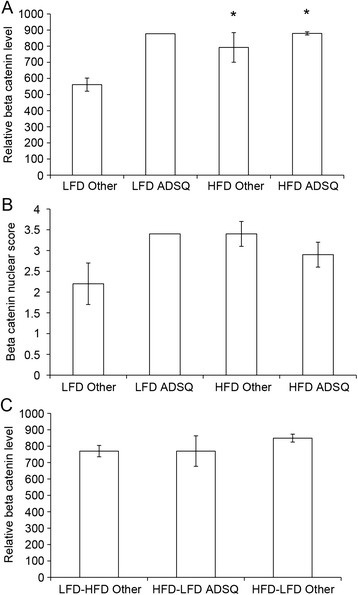
β-catenin regulation by diet treatment in tumors. β-catenin levels were measured based on immunofluorescence intensity or nuclear localization in adenosquamous (ADSQ) and nonadenosquamous (Other) tumors. **a** β-catenin levels were increased in nonadenosquamous tumors from HFD-fed mice (HFD Other) compared with those from LFD-mice (LFD Other). *p < 0.05. **b** These same tumors from HFD-fed mice (HFD Other) showed a trend toward increased nuclear β-catenin compared with nonadenosquamous tumors from LFD-fed mice (LFD Other) (*p* = 0.09). **c** Tumors from HFD-LFD- and LFD-HFD-fed mice had similar β-catenin levels. *Bars* represent mean ± standard error of the mean (n = 4–8 tumors per diet treatment). *HFD* high saturated animal fat diet, *HFD-LFD* mice on high saturated animal fat diet switched to low-fat diet, *LFD* low-fat diet, *LFD-HFD* mice on low-fat diet switched to high saturated animal fat diet

Analysis of β-catenin immunofluorescence in tumors (Fig. 7) showed similar levels in adenosquamous tumors arising in continuous LFD or HFD mice with regard to overall intensity and nuclear staining scores (Fig. 7a, b). For all other tumor types (i.e., glandular, cribriform, papillary, ductal), β-catenin levels were significantly higher in the continuous HFD group (*p* < 0.05) compared with the continuous LFD group and exhibited an increased nuclear score that approached statistical significance (*p* = 0.09). In HFD-LFD and LFD-HFD tumors, β-catenin immunofluorescence levels were similar and comparable to continuous HFD tumors, regardless of histopathological type (Fig. 7c). There were no differences in their nuclear staining scores (data not shown). Elevated β-catenin appeared to be positively associated with adenosquamous tumors, regardless of diet, and HFD exposure at any life stage, whether peripuberty or adulthood, resulted in elevated β-catenin across the various nonadenosquamous tumor histopathologies.

### Dietary effects on metabolic parameters, and systemic factors

As shown in Additional file 4: Figure S1, increased body weight was observed in the LFD-HFD mice, most notably starting at 23 weeks of age. This weight gain averaged 10 % over that in other dietary regimens. This is in contrast to the previous finding that continuous HFD initiated in peripuberty did not increase body weight [6]. However, it is noteworthy that, despite the increase in body weight, this did not promote tumor development as measured by increased incidence, decreased latency, or increased tumor cell proliferation in the LFD-HFD tumors (see Figs. 1 and 2). Additionally, despite the increase in body weight observed in the LFD-HFD mice, there were no significant differences in blood glucose or insulin levels between tumor-bearing HFD-LFD and LFD-HFD mice. Analysis of blood glucose and insulin levels showed that there were no significant differences between diet regimens at 4 weeks after diet switches (Additional file 5: Figure S2).

### Interactions between peripubertal HFD and peripubertal DMBA exposure

**Fig. 8:**
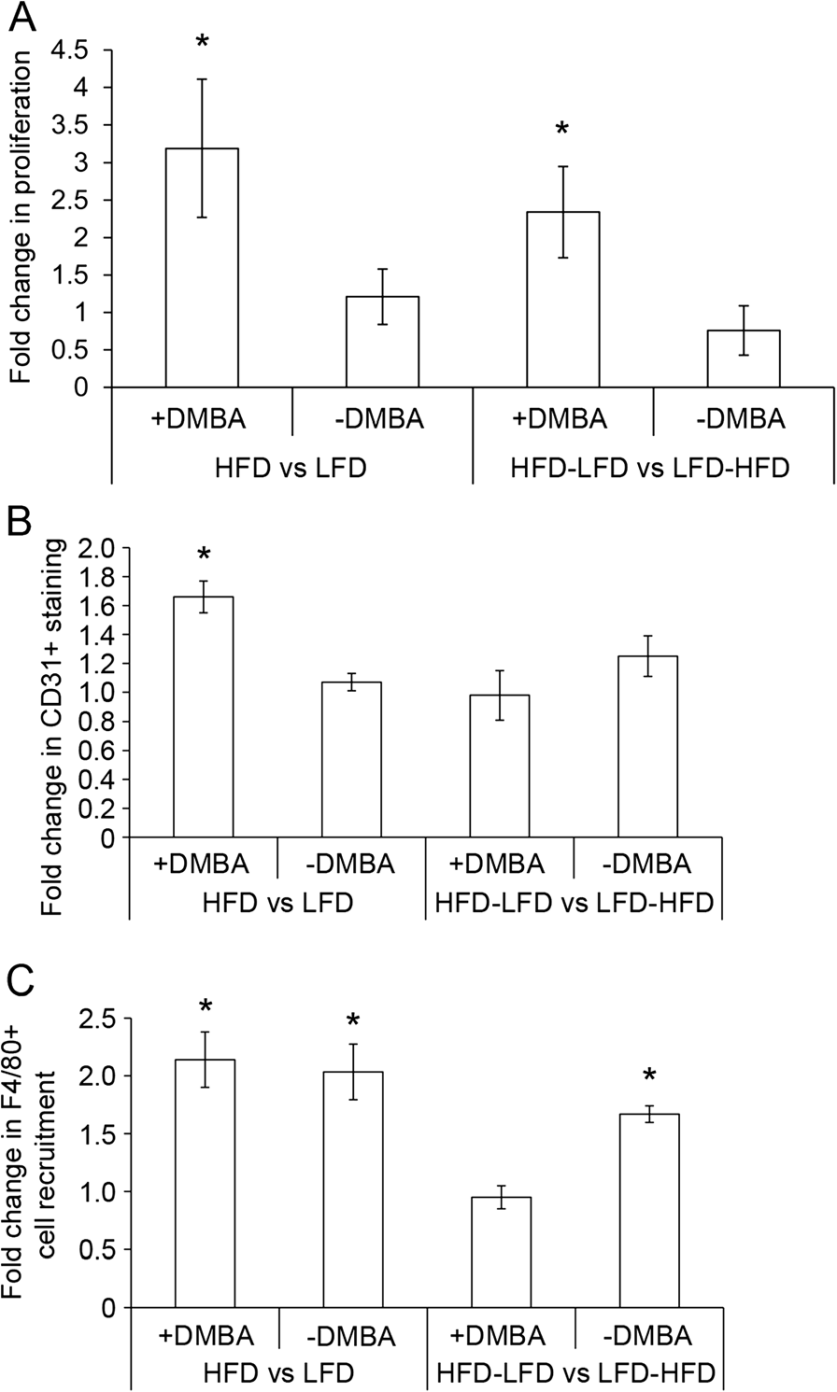
Effects of diet with or without 7,12-dimethylbenz[*a*]anthracene (DMBA) treatment in the mammary gland at 13 weeks of age. **a** Proliferation in mammary glands from mice fed HFD, LFD, HFD-LFD, or LFD-HFD was measured by 5-bromo-2′-deoxyuridine incorporation. Only diet treatments with DMBA increased proliferation (*p < 0.05). **b** Only HFD with DMBA increased blood vessel density (CD31 staining) compared with all other diet treatments (*p < 0.05). **c** HFD with or without DMBA and HFD-LFD without DMBA increased macrophage recruitment (F4/80 staining) compared with LFD and LFD-HFD without DMBA. *p < 0.05. *Bars* represent mean ± standard error of the mean (n = 5 mice per diet and DMBA treatment). *HFD* high saturated animal fat diet, *HFD-LFD* mice on high saturated animal fat diet switched to low-fat diet, *LFD* low-fat diet, *LFD-HFD* mice on low-fat diet switched to high saturated animal fat diet

We considered the possibility that some of the observed HFD effects were the result of interactions between diet and peripubertal carcinogen exposure. To examine this possibility, we analyzed the peripubertal effects of HFD in the absence of carcinogen treatment. We previously reported alterations in proliferation, immune function, and gene expression at 3 and 4 weeks of exposure to HFD in the absence of DMBA treatment [6]. At 10 weeks on diets without DMBA treatment, there were no significant differences in mammary epithelial cell proliferation for continuous HFD vs. continuous LFD or HFD-LFD vs. LFD-HFD (Fig. 8a). No significant effects on blood vessel density were observed comparing HFD-LFD mice with LFD-HFD mice that were not treated with DMBA (Fig. 8b). As noted above (Fig. 5d), DMBA-treated mice exposed to HFD over their life course had increased vascularity compared with mice with continuous LFD exposure. The overall blood vessel density was higher in the mammary glands of mice that did not receive DMBA treatment than in those that were treated with DMBA (data not shown). In the absence of DMBA treatment, macrophage recruitment was significantly increased in HFD-LFD mammary glands and similar to that in animals fed continuous HFD, whereas LFD-HFD mammary glands did not show increased recruitment (Fig. 8c). Macrophage recruitment was generally lower in the absence of DMBA treatment under all diet conditions (data not shown). As noted above (Fig. 5c), DMBA-treated mice exposed to HFD at peripuberty, adulthood, or throughout their life course exhibited increased macrophage recruitment. The overwhelming majority of macrophages under all conditions were Arg1-positive, indicating a preponderance of M2 alternatively activated macrophages (data not shown). Collectively, these data suggest that pubertal exposure is critical for macrophage recruitment. Furthermore, these results show that increased macrophage recruitment was a result of HFD exposure itself and not a result of diet interaction with carcinogens.

When we analyzed the effects of diet on gene expression in the pretumor mammary glands (13 weeks of age) of mice that had been treated with DMBA or untreated, we found that none of the genes upregulated by HFD in DMBA-treated mice (Table 5) showed significant changes in gene expression in untreated mice. This suggests that the gene expression changes that we observed in pretumor animals were the result of interaction between HFD and prior DMBA treatment.

**Table 5:** Effect of diet alone vs diet + DMBA on gene expression at 13 weeks old

		Fold regulation (compared with LFD)	
Symbol	Description	HFD + DMBA	HFD alone
*Tgfa*	Transforming growth factor alpha	2.4	NS
*Ccl1*	Chemokine (C-C motif) ligand 1	3.8	NS
*Ccl17*	Chemokine (C-C motif) ligand 17	2.8	NS
*Ccl20*	Chemokine (C-C motif) ligand 20	11	NS
*Ccl22*	Chemokine (C-C motif) ligand 22	4	NS
*Tgfb1*	Transforming growth factor, β1	1.6	NS

*DMBA* 7,12-dimethylbenz[*a*]anthracene, *HFD* high saturated animal fat diet, *LFD* low-fat diet

*p* < 0.05 for all genes listed, except NS = no significant change (n = 4 mice per diet treatment)

**Fig. 9:**
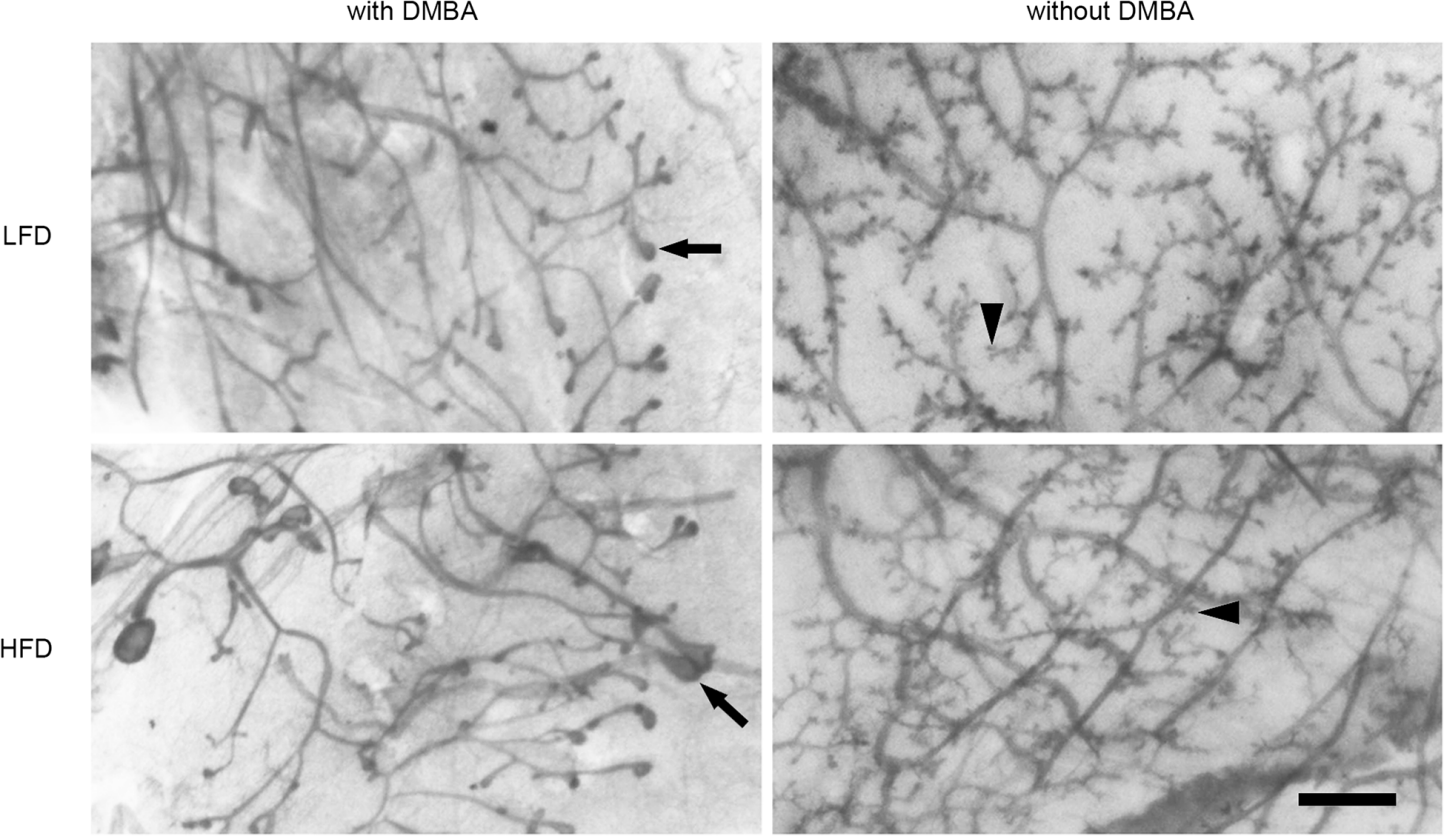
Effects of high saturated animal fat diet (HFD) with and without 7,12-dimethylbenz[*a*]anthracene (DMBA) on mammary gland morphology. Mammary gland morphology was assessed from whole mounts of mice fed HFD or a low-fat diet (LFD) with or without DMBA from 3 to 13 weeks of age. Mammary glands from either DMBA-treated HFD- or LFD-fed mice retained a pubertal ductal organization with numerous terminal end buds present (indicated by *arrows*). In the absence of DMBA, HFD- and LFD-fed mice exhibited a mature morphology indicated by extensive ductal branching and presence of ductal side branches (indicated by *arrowheads*). Representative photomicrographs of five mice per diet and DMBA treatment are shown. Scale bar = 1 mm

We also examined the effect of diet on mammary gland morphology of DMBA-treated and untreated mice at 10 weeks on diet (13 weeks of age) (Fig. 9). There was a striking difference in overall morphology. In DMBA-treated mice, the presence of terminal end buds along with reduced branching of the ductal tree was seen in the adult 13-week-old mammary glands. This was true for both LFD and HFD mice. Hyperplasia was also noted in HFD mammary glands. In contrast, both LFD and HFD mice without DMBA treatment displayed well-developed mammary glands with extensive ductal branching and side branch development indicative of mammary gland maturation (Fig. 9). There was no specific effect of diet itself on mammary gland morphology, and thus the differences appeared to be due to DMBA treatment.

## Discussion

In this study, we showed that HFD restricted to peripuberty reduced the latency of DMBA-induced mammary tumors and led to tumors with characteristics very similar to those occurring in mice fed a continuous HFD. Notably, the tumors occurring after HFD limited to peripuberty shared characteristics with basal-like human breast cancers. The incidence of basal-like, triple-negative, adenosquamous tumors was significantly increased among the short-latency tumors. On the basis of genomic and immunohistochemical analysis, adenosquamous human breast carcinomas are a rare variant of basal-like carcinomas [16]. It is notable that the incidence of adenosquamous tumors in young, ovary-intact mice temporally parallels the increased occurrence of human basal-like breast cancer in younger women [3]. The short-latency tumors showed elevated rates of proliferation, increased numbers of macrophages, and enhanced vascularization. Increased proliferation, increased hyperplasia, and increased numbers of macrophages were observed in mammary glands before the occurrence of tumors, implicating these characteristics as plausible effectors of tumor promotion as a result of peripubertal HFD exposure.

### Pubertal window of susceptibility

HFD restricted to peripuberty (HFD-LFD) reiterates the effects of a continuous HFD (HFD) and points to a pubertal window of susceptibility for HFD promotion of tumorigenesis. The short-latency tumors observed in the present study with HFD-LFD were similar to tumors that developed with HFD [6] with regard to histopathology. Both groups of tumors (HFD and HFD-LFD) were predominantly epithelial in origin, with a significant adenosquamous component, and were triple-negative (ER−PR−Her2−), similar to a subset of basal-like human breast cancers. It is noteworthy that switching to LFD in adulthood does not reverse peripubertal HFD-enhanced tumorigenesis. Furthermore, no promotional effect was observed in LFD-HFD mice, despite the long duration of HFD exposure, approaching that of continuous HFD. The data collectively support the idea of a pubertal window of susceptibility to HFD for its most profound effects on tumorigenesis and that those effects are most profound on basal-like, adenosquamous tumors.

### HFD and tumor latency

Apart from adenosquamous tumors, all other histotypes also had shorter latency in HFD mice than in LFD mice. Glandular, cribriform, and papillary tumors did not show reduced latency in HFD-LFD mice and had a similar incidence in mice on continuous HFD and LFD. Because both continuous HFD and LFD-HFD mice received quite lengthy HFD exposure, the length of treatment is unlikely to be the explanation for this difference in latency for nonadenosquamous tumors in HFD mice. Rather, the reduced latency of nonadenosquamous tumors in HFD mice is likely dependent upon a puberty-specific effect of HFD that additionally requires adult HFD exposure to promote these tumors. We speculate that the increased proliferation observed in normal mammary epithelium of both continuous HFD and HFD-LFD mice, coupled with elevated angiogenesis that is observed only in continuous HFD, is responsible. In our earlier studies [6], we found that *Vegfa* expression was increased in the mammary glands of mice fed continuous HFD, and this may play a role in elevated angiogenesis.

Interestingly, LFD-HFD tumors showed trends toward increased angiogenesis and macrophage recruitment that did not reach statistical significance, which suggests that HFD exposure limited to adulthood also affected these tumor characteristics, although to a lesser extent than peripubertal HFD exposure and not translating to increased tumor incidence or reduced tumor latency.

### Gene expression characteristics of tumors

qRT-PCR showed similar patterns of gene expression in both early HFD-LFD and HFD tumors, in accord with their similar phenotypic characteristics. Regulation of the same genes was observed across adenosquamous and epithelial histotypes. Microarray analysis also strongly supported the similarity of early HFD and HFD-LFD tumors, showing that all of these tumors cluster together, in contrast to tumors from mice on other dietary regimens and/or tumors that occur with longer latency. Notably, only mice exposed to HFD during puberty developed early tumors. Interestingly, although peripubertal HFD particularly promoted the occurrence of adenosquamous tumors, the gene clusters associated with early tumors in the microarray analysis were drawn from epithelial as well as adenosquamous tumors. Indeed, epithelial carcinomas also showed reduced latency in mice fed continuous HFD. Only one late-occurring tumor clustered with an otherwise uniform collection of early tumors. This tumor had an adenosquamous phenotype, whereas two other adenosquamous tumors clustered with late tumors. It is possible that the adenosquamous phenotype contributes to the observed early signature, but the uniform clustering of early tumors vs. the more diverse clustering of adenosquamous tumors suggests a temporal rather than a histological signature for this cluster. Collectively, the data from qRT-PCR of specific RNAs and from microarrays are most consistent with a gene expression signature for early-occurring tumors, regardless of histology. It remains to be determined whether the observed pattern of gene expression is reflective of peripubertal HFD or early occurrence of the tumors.

Examination of the existing literature reveals that, in addition to DMBA-induced mammary tumors, other mouse models that show a similarly high presentation of squamous-like mammary tumors are the *Brg1*^*+/−*^ [17] and *Pik3ca*-H1047R [18] models, the squamous tumors of which were shown by gene set analysis to be similar to claudin-low human breast tumors [19]. It remains to be determined whether the adenosquamous tumors identified in our study are similar to claudin-low human breast tumors in their pattern of gene expression.

HFD and HFD-LFD tumors showed significant similarities with regard to increased proliferation, increased angiogenesis, and increased macrophage recruitment, which indicates that peripubertal HFD treatment had a lasting effect on tumor phenotype. Consistent with enhanced proliferation, Ingenuity Pathway Analysis of the upregulated microarray gene cluster associated with the early occurring HFD and HFD-LFD tumors highlighted canonical pathways associated with proliferative processes (i.e., G_1_/S checkpoint regulation, G_2_/M DNA damage checkpoint regulation, cyclins and cell cycle regulation, antiproliferative role of TOB in T cell signaling, mTOR signaling, purine nucleotides de novo biosynthesis II, cell cycle control of chromosomal replication, and molecular mechanisms of cancer). Also consistent with enhanced proliferation, downregulated pathways associated with suppressing proliferation and/or increasing apoptosis of breast cancer cells (i.e., FXR/RXR activation and LXR/RXR activation) [20–22] were also identified. Other downregulated pathways are consistent with anti-inflammatory processes (i.e., coagulation system and acute-phase response signaling). The early HFD tumors showed higher levels of Arg1-positive macrophages [6], indicative of M2 or alternative anti-inflammatory activation. Although early HFD-LFD tumors did not show elevated levels of M2 macrophages, these pathways were not as robustly downregulated in those tumors. Additionally, pathways associated with genotoxic stress (i.e., GADD45 signaling, DNA damage-induced 14-3-3σ signaling, and EIF2 signaling) were identified among upregulated genes. This may be associated with DNA damage resulting from exposure to the mutagenic carcinogen DMBA.

qRT-PCR showed upregulation of *Ntf3*, *Trp53*, *Ccnd2*, *Ctnnb1*, *Brca1*, *Apaf1*, and *Bmp7* RNAs and downregulation of *Bmp10* RNA in both early HFD and early HFD-LFD tumors. We previously found that *Trp53*, *Bmp7*, *Ctnnb1*, and *Bmp10* identified Ingenuity canonical pathways for basal cell carcinoma signaling and role of NANOG in mammalian embryonic stem cell pluripotency [6]. This suggests that the similarity between HFD early tumors and basal-like breast cancer [23] is reiterated in the HFD-LFD early tumors. In our prior studies [6], *Trp53*, *Ctnnb1*, Ccnd2, *Brca1*, and *Apaf1* also identified Ingenuity canonical pathways for p53 signaling and GADD45 signaling. This confirms our findings in Ingenuity Pathway Analysis of the microarray clusters. *Trp53*, *Ctnnb1*, *Bmp7*, *Bmp10*, *Ccnd2*, *Apaf1*, and *Brca1* additionally identified the Ingenuity canonical pathway for molecular mechanisms of cancer, again confirming our findings from Ingenuity Pathway Analysis of the microarray clusters.

*Ntf3* and its receptor *Ntrk3*, though they do not identify a canonical pathway, are overexpressed in a significant proportion of human breast cancers, particularly in basal-like breast cancers (11 % amplified + upregulated in basal-like PAM50 breast cancers vs. 5 % in all breast cancers, 6 % in luminal A/B, and 0 % in Her2+) [24, 25]. Neurotrophins and the p75 neurotrophin receptor are expressed in human breast cancers and are implicated in promoting angiogenesis, tumor growth, invasion, resistance to apoptosis, and resistance to anoikis in triple-negative breast cancer [26–28]. Interestingly, neurotrophin expression is increased in the brains of mice fed 60 % HFD, suggesting its upregulation in early-occurring continuous HFD and HFD-LFD tumors may also be diet-induced [29].

The increased expression of *Ccnd2* (cyclin D2) is discordant with enhanced tumorigenesis of early continuous HFD and HFD-LFD tumors, as loss of cyclin D2 expression is frequent in breast cancers [30] and cyclin D2 has been considered to be a tumor suppressor. However, transgenic overexpression of cyclin D2 does block lobuloalveolar development [31], and perhaps *Ccnd2* overexpression in our system could be viewed as suppressing differentiation. It is noteworthy that *Ccnd2* expression may be specifically elevated in poorly differentiated breast cancer cells that exhibit features of epithelial-mesenchymal transition and a higher potential for metastasis [32]. Examination of The Cancer Genome Atlas database revealed that *Ccnd2* expression was altered (mainly amplified or upregulated) in 16 % of basal-like PAM50 breast cancers vs. altered (mainly downregulated) in 4 % of luminal A/B and 0 % in Her2+ breast cancers [24, 25].

In regard to increased *Ctnnb1* (β-catenin) expression, the Wnt/β-catenin pathway is involved in normal mammary gland proliferation and development and is associated with poor prognosis in breast cancer [33]. Elevated *Ctnnb1* expression may activate this pathway. We found that β-catenin protein and activation levels were elevated in all adenosquamous tumors regardless of diet, and thus this is unlikely to be a factor in their shortened latency on continuous HFD and HFD-LFD. β-catenin levels were also elevated in the nonadenosquamous continuous HFD tumors, as well as in nonadenosquamous switched-diet tumors. Thus, elevated β-catenin is associated with adenosquamous tumors regardless of diet, and HFD exposure in any life period results in elevated β-catenin across the various other nonadenosquamous tumor histopathologies. Because both HFD-LFD and HFD elevate β-catenin but HFD-LFD does not shorten the latency of nonadenosquamous tumors, it is unlikely that β-catenin is key to driving shortened latency in the nonadenosquamous tumors from HFD mice. As mentioned above, increased angiogenesis is a more likely mechanism, as it requires continuous HFD exposure.

### Characteristics of the mammary gland before the occurrence of tumors

We previously reported that, before tumor development, continuous HFD mice exhibited increased proliferation in normal mammary gland structures and hyperplastic lesions, as well as increased incidence of abnormal hyperplasia, associated with increased tumorigenesis [6]. The pretumor mammary glands of HFD-LFD mice were similar in all of these characteristics, and these factors were all likely important contributing factors in promoting tumor development after peripubertal HFD exposure. In contrast, macrophage recruitment was increased in all treatment groups that received HFD, regardless of timing, whereas increased angiogenesis required continuous HFD exposure. These latter results suggest that, if macrophage recruitment plays a role in HFD tumor promotion, it is likely through interaction with an effect specific to peripubertal exposure or with a property of the gland at this stage of development. With regard to angiogenesis, only continuous exposure to HFD was sufficient for this, and thus it was not a contributing factor to the peripubertal HFD promotional effects.

Gene expression analysis in pretumor mammary glands showed that all growth factors and chemokines observed to be elevated before tumor development in continuous HFD mice at 13 weeks of age (i.e., *Tgfa*, *Ccl1*, *Ccl17*, *Ccl20*, *Ccl22*, and *Tgfb1*) were elevated in LFD-HFD mammary glands at this time. Because LFD-HFD mammary glands do not develop early tumors, this indicates that these factors are unlikely to be factors specific to peripubertal exposure or to be responsible for the enhanced proliferation observed in normal HFD-LFD mammary glands. Only *Tgfa* was elevated in HFD-LFD mammary glands at a time after the switch to LFD. If increased *Tgfa* levels were sustained throughout the tumorigenesis period (up to 45 weeks of age) in the HFD-LFD group and did not require continued HFD exposure, this could explain, at least in part, the promotional effect of limited peripubertal exposure to HFD. *Ctnnb1* (β-catenin) expression was also increased in pretumor mammary glands of HFD-LFD mice and showed a trend toward increased expression in continuous HFD mice, indicating another early effect of peripubertal exposure to HFD. It is noteworthy that TGFα can activate β-catenin [34]. This further suggests a plausible role for TGFα in HFD-enhanced proliferation. However, TGFα itself is certainly not sufficient to promote proliferation, as elevated *Tgfa* expression and proliferation are dissociated in mice exposed to HFD only in adulthood (i.e., LFD-HFD). It may be that peripubertal HFD exposure induces another growth factor not assayed here, perhaps through the action of TGFα itself; that peripubertal HFD exposure induces long-lasting changes in the regulation of proliferation (e.g., epigenetic effects); or that peripubertal TGFα interacts with a specific population of cells not present in the adult gland (e.g., stem cells). The length of exposure to elevated levels of TGFα is a less likely explanation, as brief peripubertal exposure and lengthy continuous exposure to HFD elicit similar increases in *Tgfa* expression. The pubertal window of exposure seems critical regardless of whether elevated *Tgfa* expression is essential for enhanced proliferation. It is noteworthy that there was no overlap between the genes and pathways identified in pretumor mammary glands by expression analyses at 3 or 4 weeks on HFD and those identified at 10 weeks on HFD, as well as between those identified at any pretumor time point and in early HFD tumors [6].

### Interaction of DMBA and HFD

We previously reported that in the absence of DMBA, at 3 weeks on HFD, there was a significant transient increase in eosinophil recruitment to the periepithelial stroma, as well as transient increases in *Ccl3*, *Ccl24*, and *Il4* gene expression; a twofold increase in mammary epithelial cell proliferation; and robust upregulation of *Tnfs11* (receptor activator of nuclear factor κB ligand) gene expression at 4 weeks on HFD [6]. However, in the present study, in the absence of DMBA at 10 weeks on their diets (13 weeks of age), only increased macrophage recruitment was observed among continuous HFD and HFD-LFD mice. Importantly, this highlights the long-lasting effect of peripubertal HFD to cause higher levels of macrophages that are maintained after a switch to LFD. None of the genes identified as HFD-regulated in DMBA-treated mice were regulated by HFD in the absence of DMBA. Among those genes that were regulated by only HFD with DMBA treatment were *Tgfb1*, *Ccl1*, *Ccl17*, and *Ccl22*, whose products are all associated with the recruitment and function of immunosuppressive Treg cells [35–37]. Thus, the interaction of DMBA with HFD may influence tumorigenesis by immune modulation in addition to its activity as a mutagen. We also noted that DMBA, independent of diet, had a profound effect on mammary gland development. DMBA-treated mammary glands retained a pubertal morphology, evidenced by the presence of numerous terminal end buds and limited ductal growth. Because the pubertal gland is undergoing rapid proliferation, it is likely that the retention of a pubertal developmental state resulting from DMBA treatment contributed to the increased proliferative effects of HFD at 13 weeks of age, as previously reported [6].

An additional consideration regarding the interaction of HFD and DMBA is the possibility that HFD could increase the metabolism and activation of DMBA, thereby increasing the “effective dose” of DMBA. This could in part contribute to the increased tumorigenesis observed with HFD. It is also of interest to note that increasing doses of DMBA can also increase the proportion of adenosquamous mammary tumors [38]. However, it is noteworthy that the increased incidence of early tumors and adenosquamous tumors occurred mainly as a result of peripubertal exposure to HFD, indicating an important life-stage period of increased susceptibility to an HFD’s effects.

Another consideration regarding HFD effects in the present study are its potential contribution to tumorigenesis through increased caloric density. Lard is the major animal fat in our HFD, and it contributes to caloric density. Researchers in other studies have compared 45 % and 60 % lard HFD, which differ in caloric density, on tumorigenesis in C3(1)-T_Ag_ mice [39], and they found the same increases in tumorigenesis for both 45 % and 60 % lard HFD compared with 10 % LFD. This suggests that increased caloric density per se was not the only contributor to increased mammary tumorigenesis. However, regardless of caloric density, excess lard is apparently a risk factor for the mice; it may be the fat, or it may be the extra calories.

It is noteworthy that mice in the present study which were started on HFD in peripuberty did not exhibit a significant increase in body weight. Thus, HFD had a promotional effect on tumorigenesis in normal-weight mice. Interestingly, mice started on HFD in adulthood did gain significant body weight. However, despite the increase in body weight, this did not promote tumor development as measured by increased incidence, decreased latency, or increased tumor cell proliferation in the LFD-HFD tumors. Also to be considered are the metabolic consequences of HFD with regard to the development of prediabetic or diabetic conditions. In this regard, despite the increase in body weight observed in the LFD-HFD mice, there were no significant differences in blood glucose or insulin levels between diet regimens at 4 weeks after diet switches or in tumor-bearing HFD-LFD and LFD-HFD mice.

Studies on the effect of HFD on tumor development without obesity have been investigated in other mouse mammary cancer models. Results vary by age at diet initiation and by tumor model. In two studies of the effects of HFD initiated at 4 weeks of age in mice overexpressing *HER2/Neu* in the mammary gland [40, 41], HFD promoted tumor development by increasing tumor incidence without increasing tumor cell proliferation, and there was no insulin resistance or hyperinsulinemia. In contrast, *HER2/Neu*-transgenic mice fed HFD starting in adulthood showed no difference in tumor latency, incidence, or metastasis [42]. In the BALB/c 4 T1 tumor transplant model, mice were started on HFD at 4 weeks of age and tumor cells were transplanted after 16 weeks on diet [43]. Tumor weight and number of metastases were significantly increased by HFD. In contrast, there was no promotional effect when HFD was initiated in 10-week-old adult mice. The promotional effect observed when diet was started at 4 weeks of age is similar to our present results, showing an association of HFD with increased macrophage infiltration, angiogenesis, and cellular proliferation, as well as increased levels of a number of inflammatory factors.

## Conclusions

These studies importantly reveal a pubertal window of susceptibility to HFD promotion of DMBA-induced mammary carcinogenesis. Although the full range of etiologic factors that contribute to human breast cancer are yet to be determined, early life exposure to environmental carcinogens is a plausible contributing factor. Thus, the carcinogen-induced mammary cancer model allowed us to investigate the interaction of a carcinogenic mutagen and a lifestyle factor (dietary fat) on subsequent promotion of mammary tumorigenesis. Increased proliferation and increased tumor-associated macrophages are characteristics that are maintained in the short-latency tumors that arise after peripubertal restricted HFD, and thus they are plausible contributors to the promotion of tumor development observed with peripubertal HFD exposure. Interestingly, the only peripubertal HFD characteristic that we observed in the absence of DMBA was enhanced macrophage recruitment to the normal periepithelial compartment. This suggests that HFD-mediated macrophage recruitment induced during peripubertal exposure has the potential to influence future mammary tumor development across tumor etiologies beyond DMBA-induced carcinogenesis.

It is noteworthy that recent prospective human epidemiologic studies show compelling evidence for high total and saturated fat intake as a risk factor for ER + PR+ as well as HER2− breast cancer [44] and, strikingly, a strong association between the intake of red meat–derived animal fat and breast cancer risk in normal-weight, premenopausal women but not in overweight or obese women [5]. The latter findings are particularly in accord with our present study, where we found that an animal-derived HFD promoted tumor development in an obesity-resistant mouse model of breast cancer. The increased and early occurrence of adenosquamous carcinomas, a subtype of mammary cancer that resembles a subset of human basal-like breast cancer that also predominantly occurs at an earlier age than other breast cancers [3], suggests further parallels between these human and animal studies of diet-associated carcinogenesis. Future studies designed to reveal the mechanisms underlying these parallels are needed to identify potential interventions for the promotional effects of pubertal dietary exposure to animal-derived saturated fat. These studies have the potential to reveal intermediate biomarkers that may increase the ability to assess breast cancer risk and develop intervention strategies to reduce risk.

## Additional files

Additional file 1: Table S1. Compositions of the diets. (PDF 118 kb) Additional file 2: Table S2. Tumors used for qRT-PCR analysis and microarray analysis. (PDF 10 kb) Additional file 3: Table S3. Ontology analysis of short-latency tumors that cluster together. (PDF 38 kb) Additional file 4: Figure S1. Comparison of weight gains in mice fed HFD-LFD and LFD-HFD. BALB/c mice were started on HFD or LFD at 21 days old and then switched at 63 days old (9 weeks) to LFD and HFD, respectively, and continued until 329 days (47 weeks) of age. The decreased weight between 42 and 63 days was due to the response to DMBA treatment. Differences in body weight between the two diet groups were significant (*p* < 0.05) at every time point, except days 21, 107, 132, 139, and 209. (PDF 764 kb) Additional file 5: Figure S2. Effects of diet treatments on blood levels of glucose and insulin. BALB/c mice started on HFD or LFD at 3 weeks of age were switched to LFD or HFD, respectively, at 9 weeks of age. Blood levels of glucose (**a**, **c**) and insulin (**b**, **d**) were measured at 4 weeks post diet switches (**a**, **b**) or in tumor-bearing mice (**c**, **d**). The bars represent mean ± SEM for samples at 4 weeks after diet switches and from tumor-bearing mice (n = 5 for all groups). No significant differences were detected. (PDF 653 kb)

## Abbreviations

Ab: antibody
ADSQ: adenosquamous
Apaf1: apoptotic peptidase activating factor 1
Arg1: arginase 1
BMI: body mass index
Bmp: bone morphogenetic protein
Brca1: breast cancer 1, early onset
BrdU: 5-bromo-2′-deoxyuridine
Ccl: chemokine (C-C motif) ligand
Ccnd2: cyclin D2
Ctnnb1: catenin (cadherin associated protein), beta 1
DMBA: 7,12-dimethylbenz[*a*]anthracene
ER: estrogen receptor
FXR: farnesoid X receptor
H&E: hematoxylin and eosin
HFD: high saturated animal fat diet
LFD: low-fat diet
Ntf3: neurotrophin 3
LXR: liver X receptor
mTOR: mechanistic target of rapamycin
PBS: phosphate-buffered saline
PBSA: bovine serum albumin in phosphate-buffered saline
PR: progesterone receptor
qRT-PCR: quantitative reverse transcription polymerase chain reaction
RXR: retinoid X receptor
Tgfa: transforming growth factor α
Tgfb1: transforming growth factor β1
Trp53: transformation-related protein 53
