# Supplementary material for: Puberty-specific promotion of mammary tumorigenesis by a high animal fat diet
Source: Breast Cancer Res. 2015 Nov 2;17:138. doi: 10.1186/s13058-015-0646-4 (PMC4630903; doi:10.1186/s13058-015-0646-4)
Supplement: Additional file 1: Table S1. — Compositions of the diets. (PDF 118 kb) [file 13058_2015_646_MOESM1_ESM.pdf]

Table S1. Diet Compositions

Ingredients (g/100 g)		Low Fat Diet	High Fat Diet
Fat	Corn Oil	2.369	16.1498
	Lard	1.8957	31.6537
Carbohydrate	Corn Starch	54.407	8.888
	Maltodextrin	11.848	16.1498
Protein	Casein	18.987	25.8397
	L-cystine	0.2843	0.3876
Fiber	Cellulose	4.7393	6.4599
Vitamins	Vitamin Mix V10001	0.9479	1.2919
	Choline Bitartrate	0.1896	0.2584
Minerals	Mineral Mix S10026	0.9479	0.1286
	DiCalcium Phosphate	1.2322	1.6795
	Calcium Carbonate	0.5213	0.7106
	Potassium Citrate, 1 H20	1.5639	2.1318
**Energy**			
kcal density/g		3.8	5.2
% kcal	Fat	10	60
	Carbohydrate	70	20
	Protein	20	20
