# Supplementary material for: Puberty-specific promotion of mammary tumorigenesis by a high animal fat diet
Source: Breast Cancer Res. 2015 Nov 2;17:138. doi: 10.1186/s13058-015-0646-4 (PMC4630903; doi:10.1186/s13058-015-0646-4)
Supplement: Additional file 2: Table S2. — Tumors used for qRT-PCR analysis and microarray analysis. (PDF 10 kb) [file 13058_2015_646_MOESM2_ESM.pdf]

Table S2. Tumors used for quantitative reverse transcription PCR analysis and microarray analysis.

	Latency (wks)*	Histopathology	ER/PR
**LFD**	31	Cribriform carcinoma	ER-/PR-
	31	Papillary carcinoma	ER+/PR+
	34	Adenosquamous carcinoma	ER-/PR-
	39	Glandular carcinoma	ER+/PR+
**HFD** (early)	17	Ductal Carcinoma	ER+/PR+
	14	Adenosquamous/Cribriform CA	ER-/PR-
	10	Adenosquamous carcinoma	ER-/PR-
	10	Adenosquamous carcinoma	ER-/PR-
**HFD** (late)	29	Papillary carcinoma	ER+/PR+
	34	Papillary carcinoma	ER+/PR+
	35	Adenosquamous carcinoma	ER-/PR-
	45	Glandular carcinoma	ER+/PR+
**LFD-HFD**	28	Papillary carcinoma	ER-/PR-
	31	Glandular carcinoma	ER+/PR+
	37	Glandular carcinoma	ER+/PR+
	42	Glandular carcinoma	ER+/PR+
**HFD-LFD** (early)	10	Adenosquamous carcinoma	ER-/PR-
	16	Adenosquamous carcinoma	ER-/PR-
	17	Adenosquamous carcinoma	ER-/PR-
	19	Cribriform carcinoma	ER-/PR-
**HFD-LFD** (late)	26	Papillary carcinoma	ER-/PR-
	33	Glandular carcinoma	ER+/PR+
	38	Adenosquamous carcinoma	ER-/PR-

*Latency is presented as weeks post first DMBA treatment
