# Supplementary material for: Puberty-specific promotion of mammary tumorigenesis by a high animal fat diet
Source: Breast Cancer Res. 2015 Nov 2;17:138. doi: 10.1186/s13058-015-0646-4 (PMC4630903; doi:10.1186/s13058-015-0646-4)
Supplement: Additional file 3: Table S3. — Ontology analysis of short-latency tumors that cluster together. (PDF 38 kb) [file 13058_2015_646_MOESM3_ESM.pdf]

Table S3 Ontology analysis of short-latency tumors that cluster together.

Upregulated Pathways

Canonical Pathway	B-H Adjusted p-value	Ratio	Molecules
Cell Cycle: G1/S Checkpoint Regulation	6.10E-05	10/61	TP53,RBL2,HDAC2,PA2G4,CUL1,GNL3,SKP1,CDK2,SKP2, ATM
Cell Cycle: G2/M DNA Damage Checkpoint Regulation	6.10E-05	9/49	CDC25B,TP53,YWHAB,CUL1,TOP2A,SKP1,SKP2,CCNB1,ATM
Cyclins and Cell Cycle Regulation	2.01E-04	10/75	TP53,CCNA2,HDAC2,PA2G4,CUL1,SKP1,CDK2,SKP2,CCNB1,ATM
Antiproliferative Role of TOB in T Cell Signaling	1.04E-02	5/26	CCNA2,CUL1,SKP1,CDK2,SKP2
GADD45 Signaling	2.36E-02	4/19	TP53,CDK2,CCNB1,ATM
DNA damage-induced 14-3-3σ Signaling	2.36E-02	4/19	TP53,CDK2,CCNB1,ATM
mTOR Signaling	3.18E-02	11/176	EIF3F,EIF4G2,RHOT1,EIF4A3,EIF3I,RPS6KA3,RPS6KA5,EIF4A2,PRKD3, EIF4B,ATM
Purine Nucleotides De Novo Biosynthesis II	4.39E-02	3/11	GMPS,PAICS,PPAT
EIF2 Signaling	4.50E-02	10/161	EIF3F,EIF4G2,RPL28,EIF4A3,EIF3I,EIF2B3,EIF4A2,AGO1,RPL13A,ATM
Cell Cycle Control of Chromosomal Replication	4.84E-02	4/26	ORC2,ORC3,ORC5,CDK2
Molecular Mechanisms of Cancer	4.84E-02	16/354	TP53,RALA,PA2G4,AXIN1,NCSTN,CRK,CDC25B,GAB1, RHOT1,CASP8,PRKD3,SMAD1,CDK2,PRKAR1A,ATM,FZD7

Downregulated Pathways

Canonical Pathway	B-H Adjusted p-value	Ratio	Molecules
FXR/RXR Activation	4.87E-07	16/114	PPARG,APOA4,APOB,APOH,NR1H4,ABCC2,SERPINF2,APOA1,ABCB4, SLC51A,FGFR4,SERPINA1,FGA,GC,PON3,AGT
LXR/RXR Activation	4.66E-05	13/108	RXRG,APOA1,APOB,APOA4,APOH,NR1H4,APOA5,SERPINA1,GC,FGA, PON3,SERPINF2,AGT
Coagulation System	4.66E-05	8/35	F11,PLG,F12,SERPINC1,VWF,SERPINA1,FGA,SERPINF2
Acute Phase Response Signaling	3.35E-02	11/157	PLG,ITIH3,APOA1,APOH,IL6R,VWF,SERPINA3,SERPINA1,FGA,SERPINF2, AGT
