# Supplementary figures and images for: Puberty-specific promotion of mammary tumorigenesis by a high animal fat diet
Source: Breast Cancer Res. 2015 Nov 2;17:138. doi: 10.1186/s13058-015-0646-4 (PMC4630903; doi:10.1186/s13058-015-0646-4)

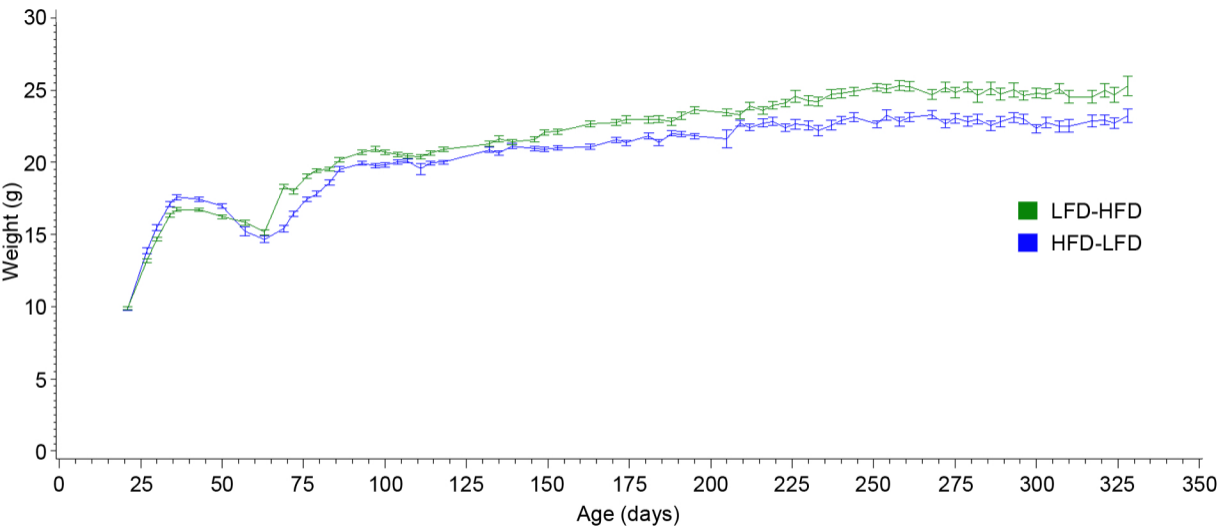

**Figure S1.** Effects of diets on body weight.

Supplement: Additional file 4: Figure S1. — Comparison of weight gains in mice fed HFD-LFD and LFD-HFD. BALB/c mice were started on HFD or LFD at 21 days old and then switched at 63 days old (9 weeks) to LFD and HFD, respectively, and continued until 329 days (47 weeks) of age. The decreased weight between 42 and 63 days was due to the response to DMBA treatment. Differences in body weight between the two diet groups were significant (p < 0.05) at every time point, except days 21, 107, 132, 139, and 209. (PDF 764 kb) [file 13058_2015_646_MOESM4_ESM.pdf]

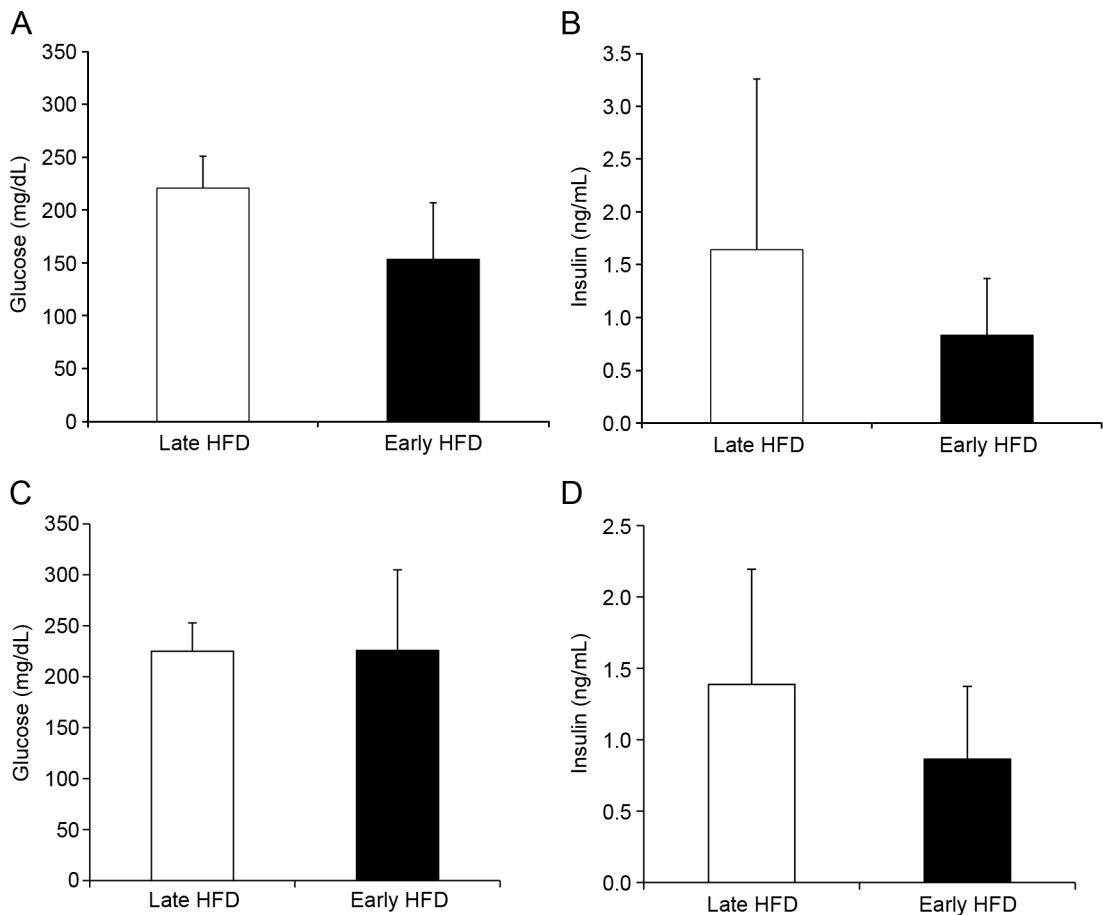

**Figure S2.** Effects of diet treatments on blood levels of glucose and insulin.

Supplement: Additional file 5: Figure S2. — Effects of diet treatments on blood levels of glucose and insulin. BALB/c mice started on HFD or LFD at 3 weeks of age were switched to LFD or HFD, respectively, at 9 weeks of age. Blood levels of glucose (a, c) and insulin (b, d) were measured at 4 weeks post diet switches (a, b) or in tumor-bearing mice (c, d). The bars represent mean ± SEM for samples at 4 weeks after diet switches and from tumor-bearing mice (n = 5 for all groups). No significant differences were detected. (PDF 653 kb) [file 13058_2015_646_MOESM5_ESM.pdf]
